# The hidden oases: unveiling trophic dynamics in Namib's fog plant ecosystem

**DOI:** 10.1038/s41598-024-61796-8

**Published:** 2024-06-10

**Authors:** Huei Ying Gan, Karin Hohberg, Clément Schneider, Martin Ebner, Eugene Marais, Tatiana Miranda, Ricarda Lehmitz, Gillian Maggs-Kölling, Hervé Bocherens

**Affiliations:** 1 Senckenberg Centre for Human Evolution and Palaeoenvironment, University of Tübingen, Hölderlindstr. 12, 72074 Tübingen, Germany; 2 Senckenberg Museum of Natural History Görlitz, Am Museum 1, 02826 Görlitz, Germany; 3 Department of Geosciences, Biogeology, University of Tübingen, Hölderlindstr. 12, 72074 Tübingen, Germany; 4 Gobabeb-Namib Research Institute, Walvis Bay, 13013 Namibia

**Keywords:** Biogeochemistry, Climate sciences, Ecology, Environmental sciences, Hydrology

## Abstract

The Namib Desert is a hyperarid coastal desert where fog is a major moisture source. We hypothesized that the fog-harvesting grass *Stipagrostis sabulicola* establishes an important ecological niche, termed the "Fog-Plant-Oases" (FPOs), and serves as the primary carbon source for the invertebrate community. To determine this, we measured the natural variations of the stable carbon and nitrogen isotopes (δ^13^C and δ^15^N) of invertebrates as well as that of plant biomass and belowground detritus and estimated the contributions of the fog plants in their diets. Our findings revealed a complex trophic structure and demonstrated that *S. sabulicola* fuels carbon flow from lower to higher trophic levels in the aboveground food web. The distinct δ^13^C values of bacterial- and fungal-feeding nematodes indicated however the separation of the aboveground niche, which is primarily sustained by *S. sabulicola*, from the belowground niche, where wind-blown sediments may serve as the main energy source for the soil biota. Our findings further accentuate the critical role of *S. sabulicola* FPOs in establishing complex trophic dynamics and a distinctive food web within the hyperarid Namib dunes.

## Introduction

Fog oases are fertile islands of vegetation that rely on fog deposition for their water supply. In botanical terms, fog oases are classified as either monospecific, consisting of a single plant species, or plurispecific, comprising multiple plant species^1^. Examples of previously described plurispecific fog oases, often at the ecosystem or community level, include the *vegetación de lomas* or coastal fog oases in Peru and Chile^2–4^, relic forests like Fray Jorge in Chile^5^, the redwood forest *Sequoia sempervirens* in northern California^6^, and cloud forests in the central South Arabian mountains^7^. Few studies have looked at monospecific fog oases; one widely examined site is the Pajonales oasis formed by the single plant species *Tillandsia landbeckii* (Bromeliaceae) in the Atacama Desert ^8,9^. Additionally, fog oases have been described at a much smaller scale, including lichen oases^10,11^ and rhizosheath of xerophytic plants^12^. From these investigations, primary motivations for studying plant oases vary, such as botanical interest and survey of plant distributions regarding geographical and climate variations. Despite these investigations, there remains a notable gap in our understanding of the ecological role of fog plants as ecosystem pioneers and the specific trophic dynamics established within these plant oases.

The trophic dynamics in an ecosystem depict its energy and nutrient transfers, commonly studied and illustrated through its food web^13,14^. Despite their major importance in global biogeochemical cycling, the soil food webs are still poorly understood, mainly due to the huge diversity of soil-dwelling and aboveground organisms of which many are general feeders^15–17^. This is exacerbated by the fact that food web studies are often focused on certain land uses and climate regions, such as agricultural lands and temperate ecosystems^15–17^. Therefore, there remains a need to continue exploring the trophic dynamics of both soil-dwelling and aboveground animal communities, particularly in arid and hyperarid ecosystems as they cover a high proportion of the Earth’s surface and due to their sensitivity to climate change^18–20^. Moreover, only a couple of studies employed stable isotope analysis in understanding the belowground food web in a hyperarid desert, including the investigation of the diet of soil nematodes and tardigrades under biological soil crusts in the arid Southwest of US^21^ and the study of the diet of nematodes, tardigrades, rotifers, and microarthropods in the McMurdo Dry Valleys in Antarctica^22^.

In this study, we examined the trophic dynamics of the monospecific fog oases established by the fog plant *Stipagrostis sabulicola* (Poaceae) in the Namib Desert, which we refer to as "Fog-Plant-Oases" (FPOs). The Namib Desert is a hyperarid coastal desert in which fog precipitation serves as a significant moisture source^23–25^. In our study sites in the Namib Sand Sea, near Gobabeb (23°34′S 15°02′E), fog from the Atlantic Ocean is a more regular moisture source (39 mm mean annual deposition) than rainfall (21.2 mm mean annual precipitation)^26^. Advection fog deposition, where the moisture is derived from evaporation over the South Atlantic Ocean and transported inland by onshore winds, is mainly influenced by the distance from the coast and elevation^27^. To cope with the extreme dryness, a considerable number of endemic species of the flora and fauna of Namib have adapted to exploit atmospheric moisture^27^. As shown previously, *S. sabulicola* possesses specialized leaf structures that are highly effective at condensing moisture from the air^28,29^. Belowground, *S. sabulicola* possesses a shallow yet extensive root system, which effectively anchors the plant in the unstable and highly dynamic windblown sand whilst facilitating the uptake of atmospheric moisture condensing on the sand surface^30^. Another important ecological role of *S. sabulicola* is in the retention of wind-blown detritus and the formation of soil hammocks or biogenic hillocks as shelter and activity centers for a high number of arthropod and vertebrate species^31,32^.

We aimed to fill in the knowledge gap by investigating the community composition and the trophic dynamics of above- and belowground invertebrates established within the *S. sabulicola* FPOs. We hypothesized that as an ecosystem pioneer, the plant biomass of *S. sabulicola* serves as the primary carbon source for the invertebrate community, which fuels carbon flow from lower to higher trophic levels. To determine this, we measured the natural variations of the stable carbon and nitrogen isotopes (δ^13^C and δ^15^N) of invertebrates as well as that of plant biomass and belowground detritus. Isotopic analysis is based on the premise that the δ^13^C and δ^15^N of above- and belowground invertebrates, to a certain degree of fractionations, reflect their diet compositions as well as feeding guilds^16,17, 33, 34^. Combined with isotope mixing models^35–37^, we aimed to estimate the contribution of *S. sabulicola* in invertebrates’ diets. Due to the importance of detritivores in organic matter decomposition in hyperarid ecosystems^38,39^, we further identified the main primary decomposers of the *S. sabulicola* FPOs and estimated the contributions of *S. sabulicola* litter in their diets.

## Results

### Diversity and abundance of FPO fauna

#### Aboveground fauna

**Figure 1:**
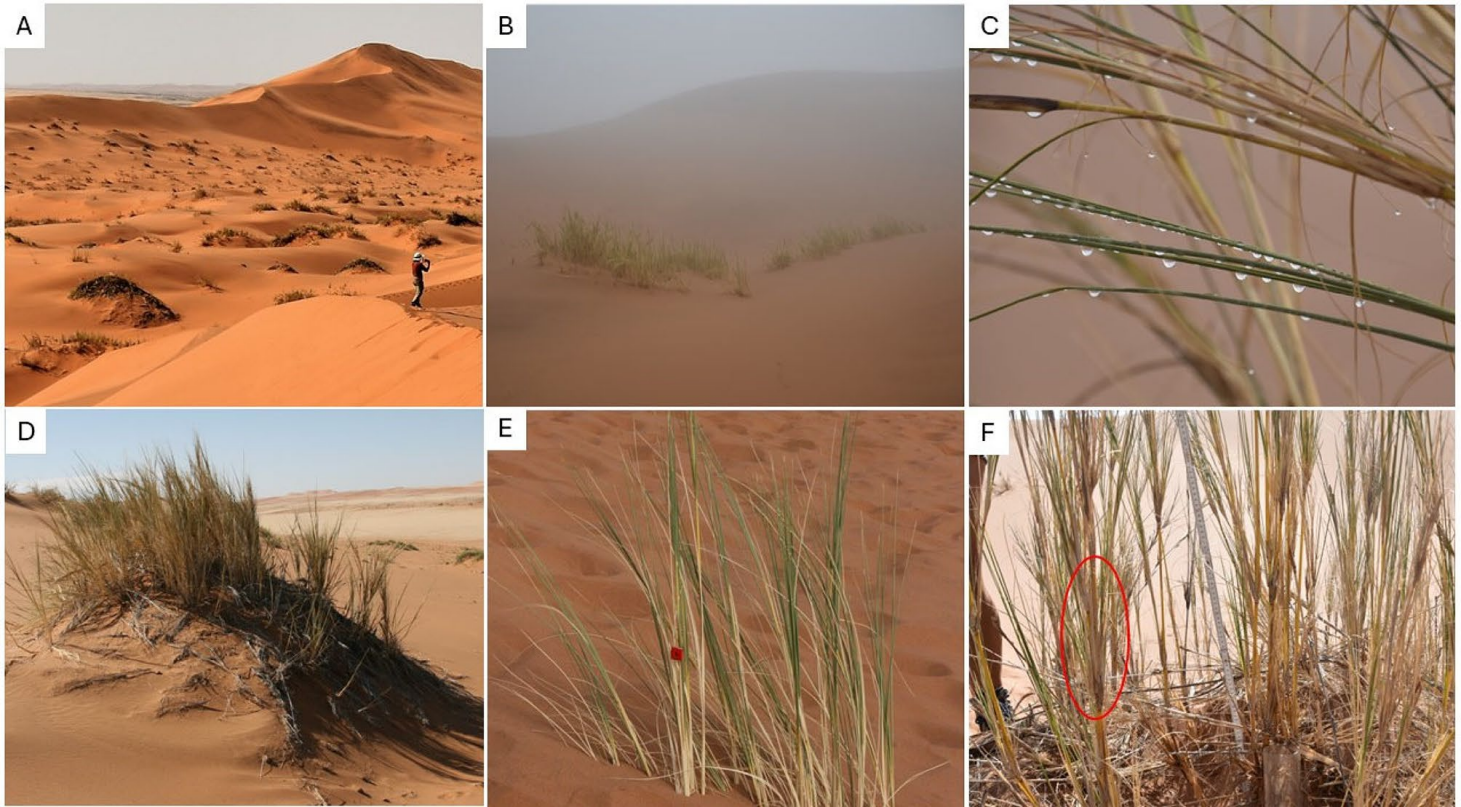
(**A**) Distribution pattern of *S. sabulicola* clusters at the station dune (**B**) Fog hitting the station dune in the early morning, (**C**) Droplets coalescing on the leaves of *S. sabulicola* due to interception of fog (**D**) Pronounced hummock underneath a cluster of *S. sabulicola* resembling an island amidst the sand sea, (**E**) “Initial” plant stages arising in a flat sand plain (**F**) Fan-structured leave bundles creating a “hidden oases” (e.g. in red circle) in the axils for aboveground arthropods.

The overall composition of non-flying or poor flying arthropods from the grass tussocks of matured FPOs was evaluated using the beating-tray technique. Rapid flyers such as Diptera and Hymenoptera often escaped and thus were mostly not studied. Collected arthropods included: oribatid mites (*Zygoribatula* sp., 100 specimens), beetles (*Cybocephalus* sp., 17 specimens, *Exochmus flaviventris*, 1 specimen), thrips (*Haplothrips* sp*.*, 15 specimens), barklice (*Liposcelis* sp., 14 specimens), weevils (*Sibinia* sp*.*, 13 specimens), pseudoscorpions (*Nanolpium* sp*.,* 9 specimens), leafhoppers (Deltocephalinae sp*.*, 5 specimens), jumping spiders (Salticinae sp*.*, 2 specimens), ground sac spiders (*Thysanina* sp*.*, 1 specimen), and parasitoid wasps (Haltichellinae sp*.*, 2 specimens). Peeling of plant stems (Fig. 1D) revealed the occurrence of armoured scale insects (Diaspididae) and mealybugs (Pseudococcidae sp*.*) and again the oribatid mite *Zygoribatula* sp*.*, under the leaf sheath near the nodes. Details on the taxonomic assignment of these species are provided in Appendix 1. Further groups observed on the leaves of *S. sabulicola*, but not studied in detail, included red velvet mites (Trombidiidae), predatory mites (Gamasina), and more insects: cockroaches (Blattodea, 1 specimen), grasshoppers (Orthoptera, 1 specimen), true bugs (Heteroptera, 1 specimen), flies (Diptera, several spp.) and parasitoid wasps (Hymenoptera, several spp.). Compared to the matured FPO sites, only a few mites were found from the tussocks and surface sand of young FPO, which were not further identified. The largest number of mites found at one site was 27 mites, together with a dead Psocodea. A table that lists the taxonomic designation of all taxa involved is included in the Appendix.

Supplementary collection of dune surface dwellers that frequently visit *S. sabulicola* included dune ants (*Campotonous detritus*), which tends scale insects and leafhoppers to collect honeydew. These ants are more abundant on the base dunes as well as on the dune slope as compared to the high dunes. Thirteen individuals of darkling beetles consisting of *Onymacris plana* (9 specimens), *Physadesmia globosa* (4 specimens) were collected both from the high dunes as well as from the dune base near the Kuiseb Riverbed. As a comparison to the *S. sabulicola* food web, two individuals of blister beetles *Hycleus zigzagus* were included, which were the herbivores of the Nara melon (*Acanthosicyos horridus*) that also occurs on the base of the dunes. *A. horridus* is a perennial endemic shrub and another potential fog-harvesting FPO that grows alongside *S. sabulicola* at the base of Namib dunes.

#### Belowground fauna

A total of 5233 nematode individuals with a total biomass of 913.4 µg biomass (fresh weight) were extracted through wet extractions (Baermann method). Most individuals were found in soils (dune sands) under mature FPOs with the highest density in deeper soil layers (30–50 cm depth). Compared to the matured FPO sites, markedly low numbers of nematodes were recovered from soils under young FPOs (a total of 3 individuals) and the open deserts next to the matured tussocks (a total of 26 individuals in 6 soil samples). The nematodes were predominantly bacterial feeders (84.8% of overall biomass) represented by the genera *Acrobeles, Cephalobus, Cervidellus, Chiloplacus, Elaphonema, Panagrobelus, Panagrolaimus, Zeldia,* and some juvenile Mesorhabditidae and Diplogastridae. Fungal-feeding nematodes contributed to 14.7% of overall nematode biomass and consisted of *Aphelenchoides, Aphelenchus, Ditylenchus, Paraphelenchus,* and a few Tylenchidae. As a third feeding group, omnivorous nematodes were detected only sporadically, but due to their large body size still accounted for 0.5% of overall nematode biomass. Tardigrades were extracted from only 3 of the 84 soil samples. They belonged to the genus *Hexapodibius* and were found with 3, 5, and 5 specimens under matured FPOs at 5–10 cm soil depth. Also, some mites (Pediculochelidae sp.) were found in very low numbers. Dry extractions (Berlese method) of another subset of 84 soil samples did not yield any animals, and only small amounts of nematodes, which were not analysed, were caught in buried pitfalls. Flotation (84 samples) yielded altogether 121 mites (Micropsammidae sp., Pediculochelidae sp., and some Astigmata (not further analysed)) and the remains of a single springtail (cuticle of an Entomobryidae).

### Stable isotope values

#### δ^13^C and δ^15^N values of basal resources

The average δ^13^C and δ^15^N values of different parts of *S. sabulicola* were summarized in Table 1. Overall, the average δ^13^C values of *S. sabulicola* spanned only 1.4‰ between − 13.7 and − 15.1‰. Larger variation was observed for δ^15^N values, which spanned almost 4‰ between 1.9‰ and 2.0‰. Compared to *S. sabulicola*, which had δ^13^C values typical of C_4_ plants, the δ^13^C values of *A. horridus* litter resembled those of typical C_3_ plants (δ^13^C = − 21.7 ± 0.8‰). Soil detritus (extracted via floatation method) under *A. horridus* (δ^13^C = − 17.1 ± 1.2‰) were markedly ^13^C-depleted compared to the dead leaves and soil detritus of *S. sabulicola*. Compared to the litter and detritus of *S. sabulicola*, the plant litter (dead stem) of *A. horridus* (δ^15^N = 4.2 ± 1.6‰) and soil detritus under *A. horridus* (δ^15^N = 3.7 ± 0.2‰) were ^15^N-enriched.

**Table 1:** Total carbon, total nitrogen, C/N ratios, and isotopic values of basal resources. Values are reported as mean ± SD.

Samples	n	Total carbon (%)	Total nitrogen (%)	C/N ratio	δ^13^C	δ^15^N
*A. Stipagrostis sabulicola*						
Fresh leaves	5	45.5 ± 1.3	1.4 ± 0.3	34.9 ± 10.4	− 15.1 ± 0.7	0.2 ± 1.5
Dead leaves	7	43.0 ± 3.0	0.5 ± 0.1	88.5 ± 20.4	− 14.0 ± 0.6	− 1.9 ± 1.6
Dead leaves with fungal infection	12	43.8 ± 2.3	0.9 ± 0.5	57.0 ± 23.1	− 14.0 ± 0.5	− 0.7 ± 2.5
Root	6	45.8 ± 0.5	0.6 ± 0.1	71.8 ± 10.1	− 14.1 ± 0.3	0.1 ± 1.4
Rhizosheath	8	20.4 ± 6.7	0.5 ± 0.2	43.5 ± 5.6	− 13.7 ± 0.4	0.8 ± 0.4
Soil detritus	7	34.5 ± 9.8	0.9 ± 0.2	38.8 ± 11.3	− 14.3 ± 0.2	2.0 ± 1.2
*B. Acanthosicyos horridus*						
Litter (Dead stem)	8	43.3 ± 3.1	1.6 ± 0.9	38.2 ± 27.6	− 21.7 ± 0.8	4.2 ± 1.6
Soil detritus	6	28.8 ± 6.6	1.1 ± 0.2	27.5 ± 9.3	− 17.1 ± 1.2	3.7 ± 0.2

#### δ^13^C and δ^15^N values of invertebrates

**Figure 2:**
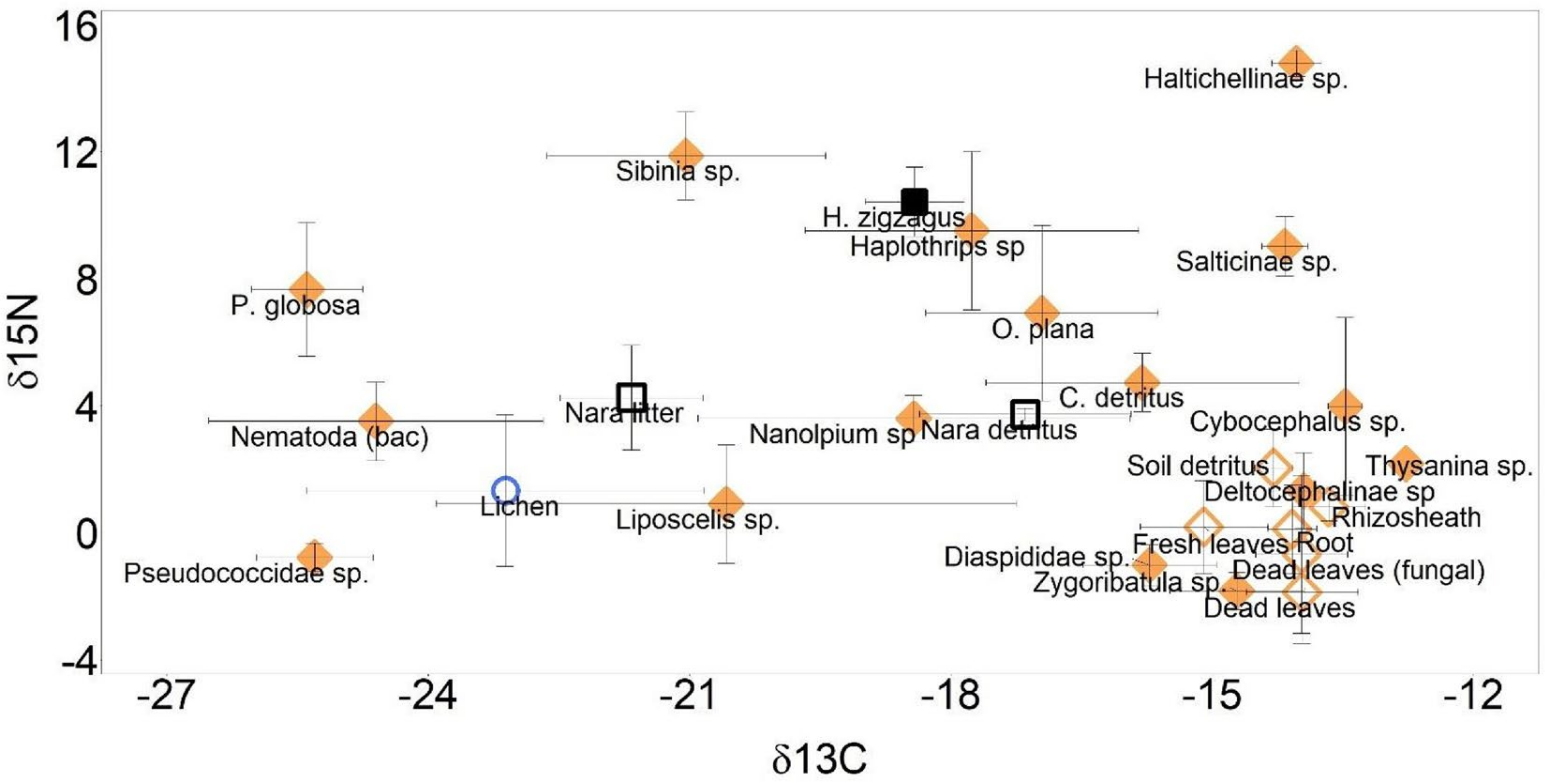
Mean (± SD) δ^13^C and δ^15^N signatures of different basal resources (open shapes) and invertebrates (filled shapes) associated with different basal resources according to different colour and shapes: sand diamonds (*S. sabulicola*), black squares (*A. horridus*), and blue circle (hypolithic biomass).

A subset of above- and belowground invertebrates were included in the stable isotope analysis for the construction of FPO trophic structure (Fig. 2). Aboveground, invertebrates that have δ^13^C values close to the dead leaves of *S. sabulicola* (δ^13^C = − 14.0 ± 0.6‰) include the saprophilous and fungivorous *Zygoribatula* sp*.* (δ^13^C = − 14.7 ± 0.7‰), sap feeders Deltocephalinae sp. (δ^13^C = − 13.9 ± 0.4‰) and Diapsididae sp*.* (δ^13^C = − 15.7 ± 0.8‰). Saprophilous and fungivorous *Liposcelis* sp*.* was markedly ^13^C-depleted (δ^13^C = − 20.6 ± 3.3‰) compared to the fresh leaves of *S. sabulicola* (δ^13^C = − 15.1 ± 0.7‰). Similarly, the herbivores *Sibinia* sp*.* (δ^13^C = − 21.0 ± 1.6‰) and *Haplothrips* sp. (δ^13^C = − 17.8 ± 1.9‰) were ^13^C-depleted compared to the fresh leaves of *S. sabulicola*. Dune surface dwellers were also ^13^C-depleted compared to the dead leaves of *S. sabulicola* including *C. detritus*: δ^13^C = − 15.8 ± 1.8‰ and *O. plana*: δ^13^C = − 16.9 ± 1.3‰*,* while the δ^13^C values of *P. globosa* were markedly depleted (δ^13^C = − 25.4 ± 0.6‰). Belowground, both bacterial- (δ^13^C = − 24.6 ± 1.9‰) and fungal-feeding nematodes (δ^13^C = − 23.6 ± 0.2‰) were markedly ^13^C-depleted compared to dead leaves of *S. sabulicola*. The summary of aboveground invertebrates, their expected trophic feeding types, and isotopic values are summarised in Table 2.

**Table 2:** δ^13^C and ∆^15^N values found for the aboveground invertebrates of S*. sabulicola* FPOs. The feeding habits from each taxa were indicated as expected diet (previous studies) and observed diet (present study).

Taxon	n	Expected diet	Source	Observed diet	δ^13^C	∆^15^N
*Zygoribatula* sp.	5 (pooled)	Saprophilous, fungivorous	^40^	Saprophilous, fungivorous	− 14.7 ± 0.7‰	0.05 ± 0.6‰
Diapsididae sp.	5 (pooled)	Sap feeder	^31^	Saprophilous/primary consumer	− 15.7 ± 0.8‰	0.9 ± 0.6‰
*Liposcelis* sp.	9	Saprophilous, fungivorous	^41^	Saprophilous	− 20.6 ± 3.3‰	2.8 ± 1.9‰
Deltocephalinae sp.	3	Sap feeder	^42^	Primary consumer	− 13.9 ± 0.4‰	3.2 ± 1.2‰
*Thysanina* sp.	1	Predator	^43^	Predator	− 12.8‰	4.0‰
*Nanolpium* sp.	4	Predator	^44^	Predator	− 18.4 ± 2.5‰	5.5 ± 0.7‰
*Cybocephalus* sp.	4	Scale insect’s predator	^45^	Saprophilous, fungivorous	− 13.5 ± 0.2‰	5.9 ± 2.8‰
*Camponotus detritus*	6	Mainly honeydew feeder	^46^	Detritivore,	− 15.8 ± 1.8	6.6 ± 0.9‰
*Onymacris plana*	8	Omnivorous, feeds mainly on plant detritus but also on green plants and dead animals	^47^	Secondary consumer/omnivorous	− 17.2 ± 1.2	7.5 ± 2.1‰
*Physadesmia globosa*	4	No specific diet, probably omnivorous	^48^	Secondary consumer/omnivore	− 25.4 ± 0.6	7.6 ± 2.1‰
*Hycleus zigzagus*	2	Flowering parts of plants	^49^	Observed to feed on Nara/omnivorous	− 18.4 ± 0.6	10.4 ± 1.1
Salticinae sp.	2	Predator	^50^	Predator	− 14.1 ± 0.3‰	10.9 ± 0.9‰
*Haplothrips* sp.	6	Herbivorous, pollinizer	^51^	Possibly predator/secondary consumer	− 17.8 ± 1.9‰	11.4 ± 2.5‰
*Sibinia* sp.	5	Herbivorous	^52^	Possibly predator/secondary consumer	− 21.0 ± 1.6‰	13.7 ± 1.4‰
Haltichellinae sp.	2	Carnivorous during larval development then nectar or honeydew feeder	^53^	Predator	− 14.0 ± 0.3‰	16.7 ± 0.4‰

**Figure 3:**
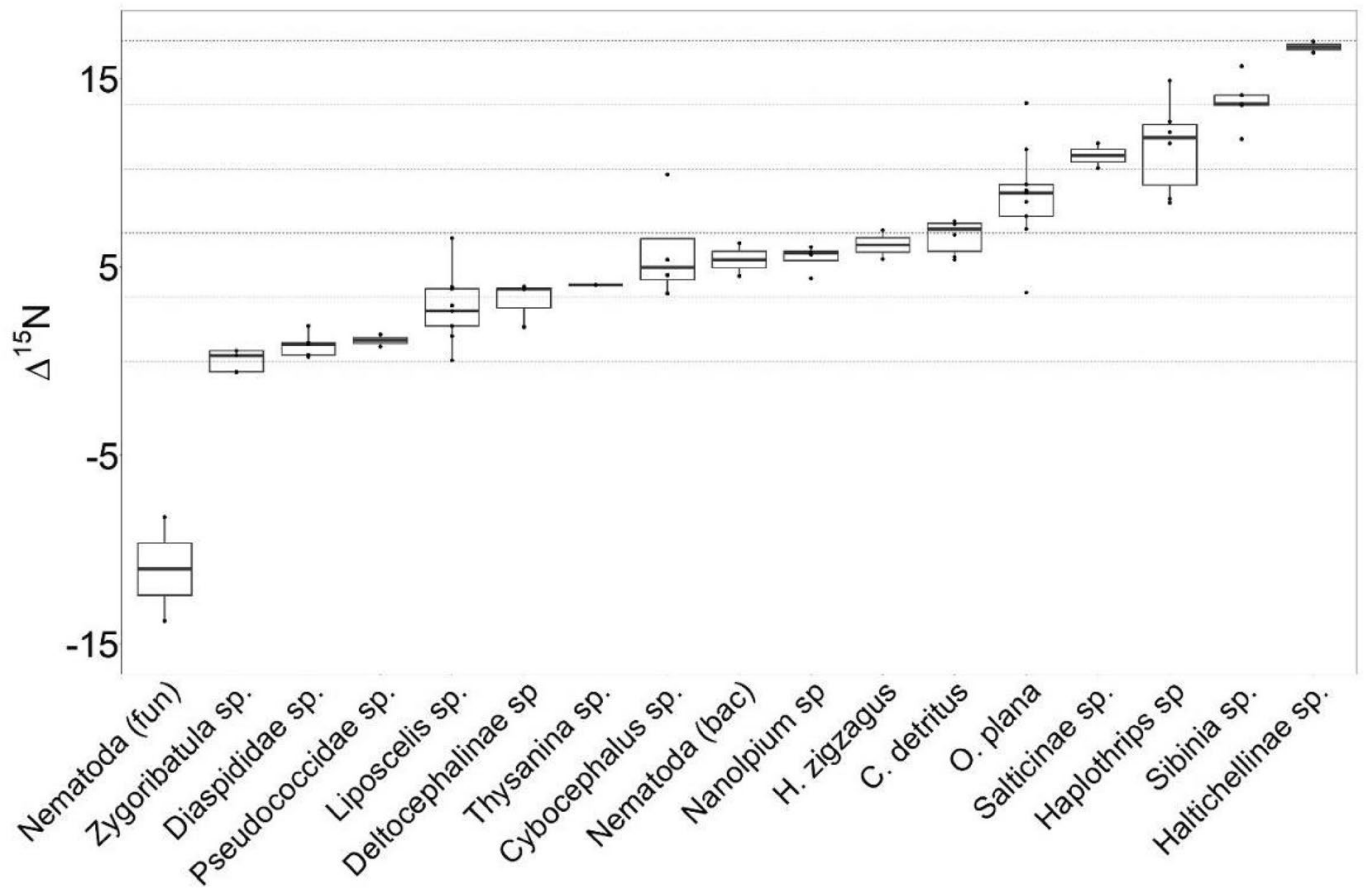
^15^N-enrichment of all above- and belowground invertebrates sampled from *S. sabulicola* FPOs as well as one species (*H. zigzagus*) from *A. horridus*. signatures.

The trophic level of the studied taxa was determined based on their bulk δ^15^N values to provide a preliminary estimation of their feeding guilds in comparison to previous studies. In total, the gradient covered a range of 16 δ units in ∆15N values across all FPO invertebrates, indicating approximately 5 trophic levels based on an enrichment factor of 3.4‰ per trophic level (Fig. 3).

#### Diet estimates of FPO invertebrates

**Figure 4:**
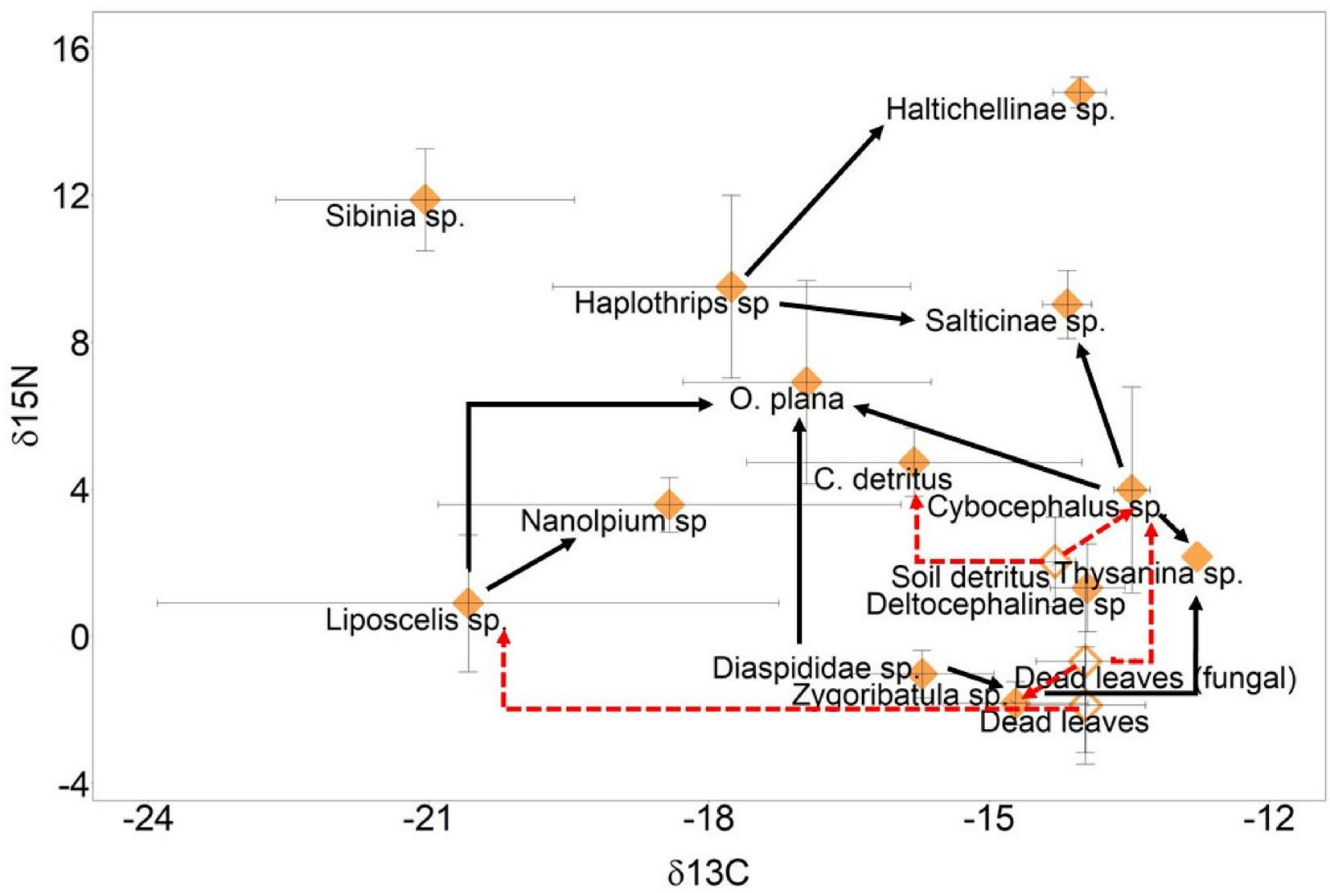
Tentative detritus and predatory food web according to stable isotope mixing models. Open diamonds indicate food sources, while filled diamonds indicate invertebrates. Dotted red arrows indicate the detrital pathway while solid black arrows indicate predation.

Estimations of potential dietary components of the sampled invertebrates assumed that invertebrates derived their energy sources primarily from *S. sabulicola*. Estimates varied markedly as indicated by high standard deviations (Tables 3, 4). Nevertheless, the results suggested that fungal-infected dead leaves of *S. sabulicola* constituted a large proportion in the diet of *Zygoribatula* sp. (70 ± 35%) and *Cybocephalus* sp. (67 ± 17%), while non-infected leaf litter contributed substantially to the diet of *Liposcelis* sp. (74 ± 42%). Softscale insects (Diaspididae sp.) had substantial contributions to the diet of *O. plana* (89 ± 5%). Soil detritus which comprises light fraction organic matter under *S. sabulicola* FPOs showed an important contribution to the diet of *C. detritus* (86 ± 18%). Among predatory taxa that occupy lower trophic levels, *Liposcelis* sp. contributed 65 ± 10% to the diet of *Nanolpium* sp., while *Zygoribatula* sp. contributed 46 ± 31% to the diet of *Thysanina* sp.. Among the “higher” predatory taxa, *Cybocephalus* sp. contributed 37 ± 17% to the diet of Salticinae sp. while *Haplothrips* sp. contributed 75 ± 10% to the diet of Haltichellinae sp.. The summary of the trophic relationships is summarized in Fig. 4.

**Table 3:** Diet estimations of detritivores by stable isotope mixing models (mean % ± SD). Mean values above 15% are marked by bold letters.

	*C. detritus*		*Cybocephalus* sp.		*Liposcelis* sp.		*O. plana*		*Zygoribatula* sp.	
	Mean	SD	Mean	SD	Mean	SD	Mean	SD	Mean	SD
Dead leaves	0.034	0.027	0.124	0.095	**0.740**	0.422	0.034	0.026	0.079	0.133
Fungal-infected dead leaves	0.033	0.026	**0.668**	0.169	0.007	0.005	0.032	0.023	**0.703**	0.349
Soil detritus	**0.856**	0.179	**0.174**	0.156	0.009	0.007	0.041	0.032	0.034	0.027
Softscale insect (Diaspididae sp.)	0.077	0.165	0.034	0.024	**0.247**	0.420	**0.892**	0.050	**0.186**	0.313

**Table 4:** Diet proportions of predators estimated by stable isotope mixing models (mean % ± SD). Mean values above 15% are marked by bold letters.

	*O. plana*		*Nanolpium* sp.		*Thysanina* sp.		Salticinae sp.		Haltichellinae sp.	
	Mean	SD	Mean	SD	Mean	SD	Mean	SD	Mean	SD
*Liposcelis* sp.	**0.452**	0.069	**0.650**	0.100	0.056	0.086	0.061	0.048	0.034	0.028
Diaspididae sp.	0.036	0.026	0.090	0.078	0.081	0.116	0.088	0.068	0.040	0.033
Deltocephalinae sp.	0.028	0.019	0.055	0.046	0.149	0.216	0.120	0.100	0.036	0.028
*Zygoribatula* sp.	0.028	0.019	0.119	0.085	**0.464**	0.307	0.078	0.058	0.033	0.025
*Sibinia* sp.	0.048	0.031	0.023	0.014	0.026	0.019	0.095	0.058	0.047	0.041
*Cybocephalus* sp.	**0.356**	0.080	0.037	0.029	**0.190**	0.252	**0.366**	0.166	0.059	0.077
*Haplothrips* sp.	0.051	0.049	0.026	0.017	0.033	0.027	**0.192**	0.133	**0.750**	0.103

## Discussion

In this study, we investigated *S. sabulicola* FPOs as a single ecosystem, encompassing both the aboveground plant tussocks and the belowground soil hammocks or biogenic hillocks (Fig. 1D). FPOs resemble islands amid the sea of sand and may comprise a single grass tussock or several conjoined tussocks (Fig. 1A). At our study sites in Gobabeb termed the “Station Dunes”, the FPO formations are predominantly monospecific on the upper dunes, characterized by the sole presence of *S. sabulicola* (Fig. 1A,B). Consistent with our observation on the Station Dunes, *S. sabulicola* has also been previously observed to be the only perennial plant species on the mid-slopes of dunes in the western dunefield^54^. Towards the less-steep plinth that extends to the interdune valley, FPOs become plurispecific, with at least two other plant species establishing such formations, including the Nara melon *Acanthosicyos horridus* and *Stipagrostis lutescens*. Another perennial succulent, *Trianthema hereroensis*, has been previously reported to be also abundant in this area together with *S. sabulicola* even during prolonged dry season^55,56^. Of these, *A. horridus* and *S. lutescens*, but not *T. hereroensis* were observed at the dune base near our study sites.

We illustrated that *S. sabulicola* FPOs harbor a diverse array of above- and belowground invertebrates. Aboveground by beating the grass tussocks, we found a total of 12 arthropod taxa on the leaf surface and within the leaf sheaths, with an additional 7 taxa observed, but not studied in detail. In contrast, flotation of soil samples yielded only a few soil microarthropod species which were predominantly mites. Belowground microarthropods under *S. sabulicola* hummock were previously recognized by Coineau and Seely^57^. They retrieved a higher diversity of taxa than us, especially with the findings of Arthropleona springtails (a single cuticle found by us). Their sampling methodology differed from ours, as they frequently watered a well in the sand for one week before sampling 10 L of sands. Our sampling design was likely not sensitive enough to capture the rare taxa (Pauropoda). From the cuticle we found, we suspect the reported Arthropleona to belong to a rather common, yet undescribed, Entomobryidae we observed in the Kuiseb and Gobabeb. The species is a rapid runner that could have rapidly aggregated in the watered well while being scarce in the natural conditions targeted by our sampling. Though beating-tray and soil samplings are difficult to compare, arthropod species richness and biomass were higher on the grass canopy of *S. sabulicola* than in the sand near the roots, revealing the complexity of these “hidden oases” (Fig. 1F). Soil life is probably inhibited by the instability of the dune surface^58^, but the presence of a thriving fauna community on the aboveground canopy was undeniably enabled by an ample supply of fog water due to stem flow and some stored in the grooved stems of *S. sabulicola*^28^. Sufficient wetting of aboveground litter has also been previously observed to undergo rapid fungal decomposition which additionally provides aboveground fauna with high-quality litter^38^. Overall, the presence of moisture, food sources, and potential wind and solar protection provided by the grass tussock canopy collectively render it a suitable habitat for the discovered invertebrates.

Previously, mostly through observations, the importance of *S. sabulicola* as a trophic hotspots and food source for several arthropods and small vertebrates has been emphasized^32,56, 59–61^. However, the use of stable isotope analysis to verify the dietary contributions of the fog plant has not yet been conducted. In this study, we showed that among the aboveground invertebrates found on the grass canopy that have similar δ^13^C values as *S. sabulicola*, their trophic groups were observed to span from the detritivore (*Zygoribatula* sp*.*) to the predatory taxa (*Cybocephalus* sp. to Salticidae sp.*,* and Haltichellinae sp.), highlighting *S. sabulicola* as the primary carbon source that fuelled the aboveground food web from the bottom to the higher trophic levels (Fig. 3). The δ^13^C values of herbivores Pseudococcidae sp*.*, *Liposcelis* sp., *Sibinia* sp., and *Haplothrips* sp. were observed to be markedly depleted compared to those of *S. sabulicola* (Fig. 2). The trend of decreasing δ^13^C values as compared to their host plant differed from earlier studies that reported a trend of increasing invertebrate δ^13^C values moving up the trophic level due to slight ^13^C enrichment^33,62^. Pseudococcidae sp*.*, which were found abundantly within the leaf folding of the *S. sabulicola* and have limited mobility (crawlers), exhibit the most distinct δ^13^C values compared to the fog plant. Therefore, it is not likely that their δ^13^C values reflect another plant’s δ^13^C values, suggesting instead that the herbivores selectively feed on specific types of photosynthetic products with depleted δ^13^C values than the bulk leaves or litter of *S. sabulicola*. Previously, variations in δ^13^C values of different primary and secondary photosynthetic products have been reported e.g. ^13^C-enriched sucrose and starch and ^13^C-depleted lignin and lipid^63^. Apart from diet, the lower δ^13^C values of these herbivores may be explained by higher lipid content in animal tissues, which is generally more ^13^C-depleted compared to proteins and carbohydrates^37^. The depleted δ^13^C values due to higher lipid content are shown by negative correlations between invertebrate C/N ratios and their δ^13^C values^64,65^. Additionally, we discovered that the FPO invertebrates showed a pattern of lower C/N ratios (Table 2) compared to soil invertebrates from temperate forests and grasslands^15,33^. We suggest that climatic influence, in this case hyperaridity, may be driving these differences.

The high δ^15^N values of Salticinae sp. (Fig. 2) indicate that they are obligatory predators, confirming previous observations that they exhibit generalist feeding habits by consuming small arthropods^66^. Moreover, their δ^13^C values, like those of *S. sabulicola*, provide evidence that their main preys occupy lower trophic levels that directly or indirectly rely on the biomass of *S. sabulicola*. Unexpectedly, diet estimates revealed a substantial contribution from fungal-infected plant litter of *S. sabulicola* to the diet of *Cybocephalus* sp., a genus of renowned scale insect predator^67^, whereas Diapsididae sp. did not exhibit a significant contribution. Whilst *Cybocephalus* sp. may feed on the fog plant for readily available water and carbon sources, the lack of direct trophic connection between the scale insect predator and its prey could be attributed to the more negative δ^13^C values of Diapsididae sp. in comparison to the fog plant (0.7‰). This renders the plant litter a more favourable resource candidate, consistent with the assumption of δ^13^C = 1.3‰ enrichment per trophic level in our model. Accordingly, higher δ^15^N values indicate that *Cybocephalus* sp. are secondary decomposers due compared to the values of primary decomposers such as *Zygoribatula* sp.. In general, a high gradient of δ^15^N (spanned 16 δ units) was observed for all above- and belowground FPO invertebrates, which spanned 5 trophic levels (Fig. 4). Using bulk δ^15^N values to determine trophic position however provides only a rough estimate, given the varying degrees of amino acid fractionation among organisms at different trophic levels^68^. Our investigations nevertheless highlight the complexity of energy flow and resource utilization within the hyperarid ecosystem.

Given the importance of detritivores in the decomposition of plant litter outside of rain events in the Namib Desert^38,39^, we further focused on the diet of FPO detritivores. We found oribatid mites from the genus *Zygoribatula* sp. to be the most abundant primary decomposer species inhabiting the leaves of *S. sabulicola.* Previous studies have reported that oribatid mites from the same genus, *Zygoribatula exilis,* exclusively occupy trees or lichens^69^. Isotope data from prior studies on *Z. exilis* in temperate forests indicated a slight ^15^N-enrichment relative to the tree barks, implying that they feed on algae and bryophyte^40^. Similarly, our diet estimates showed a majority contribution of dead leaves with heavy fungal infection to the diets of *Zygoribatula* sp. (Table 4), potentially helping to control fungal pathogens of the fog plant. Among the darkling beetles, a close resemblance of δ^13^C values between *O. plana* and those of *S. sabulicola* highlights the fog plant's importance as the primary energy source for these detritivores. Previously, *O. plana* has been observed to consume a mixture of plant- and animal-based diets but not the fog plant directly, while another *Onymacris* species, *O. laeviceps*, has been observed climbing on *S. sabulicola* to feed on its seeds^47,70^. In general, the omnivorous behavior observed in *O. plana* and *P. globosa* in this study is evident in their high δ^15^N values, similar to those of the obligate predator Salticinae sp.^66^. The diverse feeding habits and high omnivory of *O. plana* may also account for the low contribution of *S. sabulicola* biomass in their diets (Table 4), despite the potential significance of the fog plant as an energy source as indicated by their δ^13^C values. On the other hand, the δ^13^C values of *P. globosa* are significantly more negative compared to those of *S. sabulicola*, and closely resemble those of *A. horridus* at the dune base. This suggests the significance of *A. horridus* in their diets on the dune base where they were captured, or alternatively, other plants with similar δ^13^C values from the riverbed from which they are commonly found^71,72^.

The dune ants *C. detritus*, while proficient at clearing surface detritus, do not function as plant detritus consumers themselves^73^. Instead, they primarily subsist on a diet of honeydews, which are secreted by aphids and scale insects^46,73^. In line with this, we observed that the δ^13^C values of *C. detritus* closely mirrored those of the Diaspididae sp. that were notably abundant within the inner sheath of *S. sabulicola*. However, diet estimates also showed a high contribution of soil detritus in the diet of *C. detritus*, confirming the large diet spectrum of this generalist feeder. Another important detritivore associated with *S. sabulicola* is the termite *Psammotermes allocerus*, although we did not observe any during our study. These termites selectively feed on fungal-infected parts of plant litter due to their higher nutritional values^38^, a behaviour, which we observed among the oribatid mites in our study.

Few studies have investigated soil nematodes in the Namib Desert, focusing on their geographical density, functional structure, and diversity^74–76^. Like the scope of our study, these prior studies were interested in the effects of perennial plants as resource islands on the density of soil nematodes, in contrast to the open desert lacking any vegetation cover. Consistent with previous findings, we observed markedly higher soil nematode densities in the soil hammocks beneath *S. sabulicola*, especially in deeper soil layers, compared to soils without plant cover. However, we also noted markedly lower nematode densities under smaller tussocks of *S. sabulicola* lacking soil hammocks (Fig, 1E), in contrast to larger tussocks with soil hammock formations and grasses showing signs of senescence (Fig, 4D), which we refer to as the “matured FPOs” in this study. Based on their δ^13^C values, results showed a niche segregation between both bacterial- and fungal-feeding nematodes than that of the aboveground arthropods. These results suggest that while *S. sabulicola* biomass serves as an important food source for the aboveground biota, belowground, deposited atmospheric organic sources are the main energy source that sustains the soil biota. However, the degree to which the above- and belowground FPO food webs are interconnected in terms of carbon flow remains unclear, necessitating further studies. Accordingly, high δ^15^N values of bacterial-feeding nematodes indicate their trophic position as secondary decomposers, as previously proposed^77,78^. Markedly depleted δ^15^N values, however, positioned the fungivorous nematodes way below the baseline of the *S. sabulicula* plant tissues, displaying values as lichen-feeding oribatid mites in the temperate forest^40^. Results from a feeding experiment conducted by Ruess et al.^79^ showed that the depleted δ^15^N values of fungivorous *Aphelenchoides saprophilus* originated from its fungal food source that was depleted in δ^15^N values compared to the medium where the fungi grew.

Overall, we showed that *S. sabulicola* serves as the primary energy source for the aboveground food web, whereas the distinct δ^13^C values of soil nematodes emphasize the importance of deposited organic sources under FPOs as the primary food source for the belowground biota. We propose that similar trophic dynamics may occur within FPOs formed by other perennial plants like *S. lutescens* and *A. horridus*. However, due to their modified leaves into thorns, *A. horridus* unlikely exhibits the same quality of hidden oases for arthropods presented by the axils of the leaf’s bundles of *S. sabulicola*. Likewise, other microhabitats on the dune fields capable of retaining detritus, such as the slipface, do not possess the fog catalysts service provided by *S. sabulicola* and other perennial plants in this ecosystem. The trophic dynamics of the FPO, as demonstrated in this study, however, go far beyond the invertebrate food web. This is because numerous arthropods that depend on *S. sabulicola* as their source of energy and thus live closely related to the plants serve as vital food sources for more mobile invertebrate and vertebrate predators. Weevils and darkling beetles are for example well-studied prey of two closely related Namib Desert sand lizards, *Meroles cuneirostris* and *Aporosaura anchietae*^80^, to the huntsman spiders in the Sparassidae family^81^, as well as to the dune lark *Mirafra erythrochlamys*^82^. Moving up the higher food chain, the lizards are again important food for the sidewinding adder *Bitis peringueyi*^83^. In summary, our research highlights the critical function of *S. sabulicola* FPOs in supporting complex trophic dynamics within the hyperarid Namib dunes.

## Methods

### Site description

The study was conducted in the Namib Sand Sea near the Gobabeb Namib Research Institute (23°34′S, 15°03′E, 407 m a.s.l., ~ 1 km south across the Kuiseb River). *S. sabulicola* is the dominant plant species growing on the Aeolian dunes, particularly the plinth (flattened slope on the dune base) and the windward slopes^32,84^. At the dune base, *S. sabulicola* was found to coexist with *Stipagrostis lutescens* and *Acanthosicyos horridus* (Nara). A total of seven FPOs established by *S. sabulicola* with two contrasting grass tussock sizes (3 young and 4 mature tussocks with signs of senescence) were examined. Mature and young FPOs referred to growth stages of *S. sabulicola* that forms the FPO. At mature FPOs, sizable sand hummocks (up to 10 m × 9 m wide) were formed by tussocks that frequently grow up to 2 m tall, while young plants (under 1 m in height) did not accumulate visible hummocks yet. Open sand next to the FPOs were assigned as controls. All three young FPOs and two mature FPOs were located on dune ridges, while two mature FPOs were located on the windward plinth and dune base respectively.

### Sampling and processing of samples

Field work and sampling of all study materials were carried out during the fog season in early September 2022. Samples for extracting soil nematodes and other belowground invertebrates consisted of two replicates (one for wet extractions for nematodes and one for other fauna) of 3–4 soil cores each per FPO (diameter 5 cm, height 5 cm), with 84 samples in total. Immediately after sampling, nematodes were extracted from soil samples using a modified Baermann method (wet extraction) for 48 h. In this study, we used "soil" as a generic term for "ground substrate", which in this case refers to sandy substrate from the dunes. At the end of the extraction, nematodes were heat-killed and fixed with formaldehyde. The nematodes were identified to genus level, and where possible to species, under an inverted microscope (400 × magnification). Nematode genera were assigned to bacterivorous, fungivorous, and omnivorous feeding types following Yeates et al.^85^. Several adults from each genus were isolated for preparation on permanent slides and species determination. The remaining bacterivores yielded sufficient biomass for isotope analysis, hence were hand picked under the inverted microscope in two samples, which weighed 48.5 and 105.8 µg dried nematode mass, respectively. The numbers and biomass of fungivorous nematodes did not suffice, thus two samples containing both bacterivores and fungivores were weighed together. Since in both of these pooled samples, the biomass ratios of the two trophic groups (m_bac_ and m_fun_) were accurately measured, and the isotopic signal for bacterivores (F_bac_) was already determined, the isotope signals (F_fun_) could be calculated from the isotopic signal F_pool_ and biomass m_pool_ of the pooled samples, using a two pools mass-balance equation: m_pool_F_pool_ = m_bac_F_bac_ + m_fun_F_fun_, where m = the biomass ratios and F = the fractional isotopic abundance of bacterivorous and fungivorous nematodes within the two pooled samples.

Other soil arthropods were investigated using three methods: Berlese-Tullgren funnels (dry extraction), buried pitfalls, and flotation methods. Berlese-Tullgren were made by filling a metal ring with sand (80 cm^3^), with a four-time folded mosquito net 1 mm mesh size) used as a substrate retention filter, the ring and net were tightly fit to a small funnel and the above heat was provided by halogen lamps. The method proved impractical and was discontinued after we verified that 18 first samples were either empty (or accidentally filled with sand). Dry buried pitfalls were made of 20 ml plastic tubes baited either with flakes of yeast or with vegetarian fish food and a piece of wetted paper; wet buried traps were made of the same tubes but only filled with water. The tubes were closed with four layers of mosquito net and buried 5 cm under the sand, within a large *Stipagrostis* tussock. Five traps of each type (yeast, fish food, and water) were used. The sand above the traps was sprayed with water every evening, for 5 days. None of those traps yielded any results on the study site (but a similar trap was successful in capturing springtails in the dry sand of the Kuiseb riverbed). Flotation was made by pouring 80 cm^3^ of sand in tap water. The sand was resuspended 10 times with a spoon with care to minimize disturbance on the water surface. After 30 min, the water surface was observed with a stereomicroscope and floating animals were picked with a micro-spoon and transferred into pure ethanol.

The small invertebrates dwelling on the stems and leaves of the plant were collected using the beating-tray technique then captured with a mouth aspirator modified for immediate preservation in ethanol. We collected most arthropods that did not fly away immediately. The macrofauna (ants and darkling beetles) was collected on sight by hand. Stems of the plant were also peeled under the stereomicroscope to recover small invertebrates living under the leaf layers. All fauna except for nematodes were killed and preserved in 100% ethanol solution. Preservation of faunas in ethanol and formalin can affect their isotopic values but the shift in values was usually less than 1‰^86,87^. Plant materials (fresh leaves, dead leaves, plant roots, and rhizosheath) were dried at 60 °C for 3–5 days. Soil detritus was extracted using a flotation method (3:1 water:soil ratios) then filtered through a fine mesh (6 µm).

We sorted the animals into morphospecies using a stereo-microscope. We selected the morphospecies we estimated to offer enough biomass for stable isotopes analysis and molecular sequencing. One individual of each was selected for genome skimming, either from a cutted leg, or from the whole individual depending on the size. DNA extractions were done using the DNeasy Blood & Tissue Kit (Qiagen, Hilden), Illumina libraries were made using the NEBNext^®^ Ultra™ II DNA Library Prep Kit (New England Biolabs, Ipswich), for a 150 bp insert size. Sequencing was done at Novogene UK on a NovaSeq 6000 system (Illumina, San Diego), aiming for 10 Gb per library. The sequenced libraries were trimmed with Trimmomatic (v0.39)^88^ then assembled with SPAdes (v3.14.1)^89^. The COI-5P marker (658 bp) and the 28S rDNA full gene was searched directly in the scaffolds using Blastn (v2.13.0+). The integrity of the protein coding sequences was verified, and each sequence was first queried on Genbank to check for obvious contaminants. We further confirmed that none of the sequences could be directly assigned to a species using the BOLD identification tool. We then proceeded to a phylogenetic placement. We adapted our method case by case, but the general approach was as follows. For each morphospecies, we subselected the BOLD public database using the lowest taxonomic group we recognized (Araneae, Cicadellidae, Coccoidea, Cucujoidea, Hymenoptera, Psocodea, Pseudoscorpionides and Thysanoptera). For each supra-family taxa selection, we sampled the database allowing from 20 to 50 records for each family (duplicated sequences removed, records randomly selected, and a number of maximum records manually tuned to keep the number of OTUs around or below 3000). A rapid phylogenetic placement was performed using MAGUS^90^ for multiple sequence alignments and FastTree2 (Price et al., 2010) for ML tree inference. When the dataset contained less than 400 OTUs, we used Muscle v5 (Edgar, 2022) + Raxml-ng^91^ instead. The trees were visualised using iTOL^92^. The closest family was selected as a novel filter of the BOLD database, and the sampling process was repeated at genus level. For some taxa, COI-5P did not allow a taxonomic placement. In such cases we retrieved suitable comprehensive phylogenetic datasets based on 28S rDNA and used them to accurately confirm the family of the species. Then again, high resolution placement was done using the COI-5P against the BOLD public database. Once the closest genus could be identified, we searched the literature for previous reports in Namibia. Details for each species are provided in Appendix 1.

### Stable isotope analysis and statistics

Prior to weighing, all plant and detritus samples were hand-milled manually using a pestle and mortar to ensure homogeneity and transferred into tin capsules. Invertebrates were transferred directly in tin capsules and oven-dried at 60 °C for at least 24 h. Nematodes were weighed in tin capsules with lids to avoid biomass loss from evaporation. Bigger arthropods (darkling and blister beetles) were freeze-dried (Heto PowerDry LL3000; Thermo Fisher Scientific, Waltham, USA) for 48–72 h. C/N concentrations and stable isotopes were quantified using an elemental analyzer coupled to isotope-ratio mass spectrometer (IRMS) (MAT 251, Finnigan, Bremen, Germany)^93^. Isotope ratios of all samples were reported in conventional δ-notation, where R is the ratio of heavier to lighter isotope. The δ values were expressed as per mill (‰) or parts per thousand.

Values of δ^15^N in a consumer’s tissue are more ^15^N-enriched compared to those of its diet, and this is termed “enrichment” as denoted by ∆, where ∆^15^N = δ^15^N_consumer_—δ^15^N_diet_ (Eq. 1). In the present study, ∆^15^N values were used to calculate trophic levels of above- and belowground invertebrates at the *S. sabulicola* FPOs, where dead leaves of the fog plant were assumed to form the trophic base (∆^15^N = 0) of the invertebrate food web. Calculation of trophic positions assumes the enrichment factor per trophic level equals 3.4‰ according to a previous large-scale study on marine and freshwater organisms by Minagawa and Wada^94^. Therefore, Eq. 1 is rewritten as TP = [δ^15^N_invertebrate_ − δ^15^N_baseline_]/3.4, where δ^15^N_invertebrate_ = δ^15^N values of above- and belowground invertebrates found on or within proximity to *S. sabulicola*, and δ^15^N_baseline_ = dead leaves of *S. sabulicola*. Likewise, the δ^15^N_baseline_ to calculate the trophic position of *H. zigzagus*, the herbivore species found feeding on *A. horridus*, was assumed to be the δ^15^N values of dead *A. horridus* stem.

All statistical analyses were conducted in R version 3.3.2^95^. The R package Stable Isotope Mixing Models (function “simmr”) was used for the reconstruction of faunal diet^28,35^. The mixing models required both the δ^13^C and δ^15^N values of consumers as well as likely sources (diet) including corrections (i.e. standard deviations for δ^13^C and δ^15^N values, enrichment factor for ^13^C = 1.3 ± 0.4‰ and ^15^N = 3.4 ± 1.0‰ according to, as well as the % of carbon and nitrogen in diet). Mixing models were used to estimate (1) the contributions of dead leaf, soil detritus, and scale insects (Diaspididae sp.) to the diet of primary as well as secondary decomposers and (2) the contributions of lower-ranked invertebrates to the diet of predatory taxa. Fitting linear models (function “lm”) were used to determine the correlation between δ^13^C of invertebrates and their C/N ratios.

### Plant collection statement

The plant collection and use were following all the relevant guidelines provided by the Namibian National Commission on Research, Science, and Technology, as stipulated on the research permit issued with Permit Number RPIV00672022. The plant material used in this study was identified by Dr. Tatiana Miranda and Dr. Martin Ebner, both co-authors of this paper. They have previously published a paper on the same plant *S. sabulicola*.

### Supplementary Information

Supplementary Information.

## Data Availability

The animal’s DNA sequences are publicly deposited on BOLD (https://www.boldsystems.org), while voucher specimens are deposited in the collections of the National Museum of Namibia, Windhoek. Source data are provided in this paper.
