# Supplementary material for: The hidden oases: unveiling trophic dynamics in Namib's fog plant ecosystem
Source: Sci Rep. 2024 Jun 10;14:13334. doi: 10.1038/s41598-024-61796-8 (PMC11164947; doi:10.1038/s41598-024-61796-8)
Supplement: Supplementary file 1 — Supplementary Information. [file 41598_2024_61796_MOESM1_ESM.docx]

**Appendix 1: List of all taxa involved in the present study**

| **class/clade** | **order** | **family** | **genus/species** | **common name** |
| --- | --- | --- | --- | --- |
| Plant |  |  |  |  |
| Monocots | Poales | Poaceae | *Stipagrostis sabulicola* | Namib dune bushman-grass |
| Dicotyledons | Cucurbitales | Cucurbitaceae | *Acanthosicyos horridus* | Nara |
| Invertebrates |  |  |  |  |
| Insecta | Hemiptera | Diaspididae | Diapsididae sp. | armoured scale insects |
| Insecta | Hemiptera | Pseudococcidae | Pseudococcidae sp. | mealybugs |
| Insecta | Hemiptera | Cicadellidae | Deltocephalinae sp. | leafhoppers |
| Insecta | Coleoptera | Cybocephalidae | *Cybocephalus* sp. | sap/ bark/ fungus beetle |
| Insecta | Coleoptera | Tenebrionidae | *Onymacris laeviceps* | darkling beetle |
| Insecta | Coleoptera | Tenebrionidae | *Onymacris plana* | darkling beetle |
| Insecta | Coleoptera | Tenebrionidae | *Physadesmia globosa* | darkling beetle |
| Insecta | Coleoptera | Curculionidae | *Sibinia* sp. | leguminous seed weevils |
| Insecta | Coleoptera | Coccinellidae | *Exochomus flaviventris* | lady bugs |
| Insecta | Psocodea | Liposcelidinae | *Liposcelis* sp. | booklice/barklice |
| Insecta | Hymenoptera | Formicidae | *Camponotus detritus* | Namib Desert dune ant |
| Insecta | Hymenoptera | Chalcididae | Haltichellinae sp. | chalcid wasps |
| Insecta | Thysanoptera | Phlaeothripidae | *Haplothrips* sp. | thrips |
| Arachnida | Pseudoscorpiones | Olpiidae | *Nanolpium* sp. | Pseudoscorpions/ false scorpions |
| Arachnida | Oribatida | Oribatulidae | *Zygoribatula* sp. | oribatid mite/ moss mites/ beetle mites |
| Arachnida | Araneae | Trachelidae | *Thysanina* sp. | huntsman spider |
| Arachnida | Araneae | Salticidae | Salticinae sp. | jumping spider |
| Arachnida | Acariformes | Pediculochelidae | Pediculochelidae sp. | mites |
| Arachnida | Acariformes | Pediculochelidae | Micropasmmidae sp. | mites |
| Arachnida | Acariformes | Pediculochelidae | Pediculochelidae sp. | mites |
| Arachnida | Sarcoptiformes | Sarcoptidae | Astigmata | astigmatid mites |
| Chromadorea | Rhabditida | Cephalobidae | *Acrobeles* sp. | nematodes |
| Chromadorea | Rhabditida | Cephalobidae | *Cephalobus* sp. | nematodes |
| Chromadorea | Rhabditida | Cephalobidae | *Cervidellus* sp. | nematodes |
| Chromadorea | Rhabditida | Cephalobidae | *Chiloplacus* sp. | nematodes |
| Chromadorea | Rhabditida | Cephalobidae | *Zeldia* sp. | nematodes |
| Chromadorea | Rhabditida | Elaphonematidae | *Elaphonema* sp. | nematodes |
| Chromadorea | Rhabditida | Panagrolaimidae | *Panagrobelus* sp. | nematodes |
| Chromadorea | Rhabditida | Panagrolaimidae | *Panagrolaimus* sp. | nematodes |
| Chromadorea | Rhabditida | Aphelenchoididae | *Aphelenchoides* sp. | nematodes |
| Chromadorea | Rhabditida | Aphelenchidae | *Aphelenchus* sp. | nematodes |
| Chromadorea | Rhabditida | Aphelenchidae | *Paraphelenchus* sp. | nematodes |
| Chromadorea | Rhabditida | Rhabditidae | Rhabditidae sp. | nematodes |
| Chromadorea | Rhabditida | Diplogastridae | Diplogastridae sp. | nematodes |
| Chromadorea | Rhabditida | Anguinidae | *Ditylenchus* spp. | nematodes |
| Chromadorea | Rhabditida | Tylenchidae | Tylenchidae spp. | nematodes |
| Eutardigrada | Parachela | Halobiotidae | *Hexapodibius* sp. | tardigrades |
| Pancrustacea | Entomobryomorpha | Entomobryidae | Entomobryidae (single cuticle) | springtail |

**Appendix 2: information about the above-ground fauna taxonomy**

This appendix provides information about the identity of the above-ground small invertebrates (body size < 5 mm) collected on *Stipagrostis sabulicola*. While most of those species were not fully identified, this content will allow the link with species list in future taxonomic or ecological work on *Stipagrostis sabulicola* habitat. Sequencing data accession numbers to COI-5P (target region size = 658 bp) and complete 28S rDNA are provided in Table S1 of this appendix. Figures S1 – S31 are included directly at the end of this appendix. Phylogenetic trees were trimmed to show only the close phylogenetic context of each OTUs. The following ‘grey literature’ checklists of Namibian species mentioned in the text are abbreviated as follows:

NBD – The Namibia Biodiversity Database Web Site (Irish 2003-2023, consulted the 15th of October 2023).

NSSWH – Namib Sand Sea World Heritage Nomination. Annex 11. Table of Invertebrates & Protista (Seely 2012).

**ARACHNIDA**

**Araneae**

**Salticidae**

**Salticinae sp.**

Dorsally white with markings dorsally on the cephalothorax and abdomen, of variable intensity, from orange to dark brown (Figs. S1, S2). Ventrally whitish, as the legs but with dark extremities (Fig. S3). Phylogenetic placement, among 341 Salticidae OTUs (BOLD) retrieves it as the sister group of *Cembalea heteropogon* (Simon, 1910) (Fig. S4), nested within the *Heliophanus* genus. *Cembalea heteropogon* is already known from Namibia (NBD). Other non-sequenced representatives of *Cembalea* are present in Namibia: *C. hirsuta* Wesołowska,  2011 and *C. triloris* Haddad & Wesołowska, 2011 (NBD). All three species differ from our specimens from the body colour patterns (Simon 1910, Haddad & Wesołowska 2011, Wesołowska 2011). We remain cautious regarding the genus assignation, until proper morphological analysis can be conducted.

**Trachelidae**

***Thysanina* sp.**

A whitish spider with grey markings dorsally on the abdomen (Fig. S5). Phylogenetic placement, among 116 Trachelidae OTUs (BOLD) , retrieves it as sister to an unidentified *Thysanina* sp. the pair being sister to *Thysanina gracilis* Lyle & Haddad, 2006 (Fig. S6). This latter species and several other *Thysanina* spp. are known to occur in Namibia (Lyle & Haddad, 2006). Its white colour seems similar to *Thysanina serica* Simon, 1910 but the abdominal pattern differs (Lyle & Haddad, 2006).

**Oribatida**

***Zygoribatula* sp.**

Roundish, brown shelled oribatid mite (Figs S7, S8), retrieved abundantly on *S. sabulicola*. Morphological investigations were conducted allowing us to recognize an undescribed species of the species-rich genus *Zygoribatula*. The species description is pending.

**Pseudoscorpionides**

**Olpiidae**

***Nanolpium* sp.**

**?= Nanolpium sp. B : Judson & Heurtault (1996) p. 324.**

White pseudoscorpion with dark claws (Fig. S9). Found sister to *Olpium pallipes* (Olpiidae) (Fig. S10). Another Olpiidae, “*Nanolpium* sp. B” was previously reported from *S. sabulicola*, 6 km South-West of Gobabeb (Judson & Heurtault 1996). We confirmed morphologically the *Nanolpium* identity (Beier 1964, 1966). It is likely that our species is the same as the one reported by Judson & Heurtault (1996), being rather common on *S. sabulicola*, and also being the only Pseudoscorpion species we collected here.

**INSECTA**

**Coleoptera**

**Cucujoidea**

**Cybocephalidae**

***Cybocephalus* sp.**

Dark, shiny, rounded beetle, dorsally convex, with the ability to fold its head under the pronotum (Figs S11, S12). The final step of our phylogenetic placement included 377 OTUs representing seven families of Coleoptera; and retrieved this species as sister-ground to *Cybocephalus* (being the only Cybocephalidae publicly represented on BOLD). Subtree is shown in Fig. S13. Assignation to *Cybocephalus* was then confirmed by Dr. Trevor Smith, from pictures. The NBD reports only two species of Cybocephalidae, *Cybocephalus dudichi* Endrödy-Younga, 1962 and *Cybocephalus rudebecki* Endrödy-Younga, 1967, but further study is necessary to confirm our species identity. No Cybocephalidae were reported from the Namib Sand Sea to our knowledge. *Cybocephalus* species are primarily feeding on scale insects, and complement their diet with pollen (Smith 2022).

**Coccinellidae**

***Exochomus flaviventris* Mader, 1954**

Round ladybird with black elytra and yellow spots on the prothorax, the frons and the clypeus (Fig. S14). Abdominal sterna 1 to 3 ventrally mostly black, turning yellow toward the sides, abdominal sterna 4 to 6 ventrally yellow (Fig. 15). We collected a single specimen of this species, with colours matching the male of *Exochomus flaviventris* Mader (Fabres et al. 1981), which is also reported in the Namib Sand Sea (NSSWH). *E. flaviventris* is a well documented predator of mealybugs and scale insects (Fabres et al. 1981). *E. flaviventris* has been reported to be the biggest consumer of cassava associated coccids, compared to other cassava associated ladybirds (Kanika-Kiamfu et al. 1993). DNA barcode unfortunately not obtained.

**Curculionidae**

**Tychiini**

***Sibinia* sp.**

Light brown weevil (in ethanol), Fig. S16. Our rapid phylogenetic placement retrieved this species nested in *Sibinia*. The final step included 82 OTUs representing *Sibinia* and the closest genera selected from the preliminary tree (*Eubulus*, *Micrelus*, *Cleopomiarus*, *Miarus*, *Ochyromera*, *Plocetes*, *Lignyodes*). Subtree is shown in Fig. S17. Bootstrap values are low at the *Sibinia* node. We then confirmed that the species was fitting with the morphological diagnosis of *Sibinia* provided by Clark (1978): sides of abdominal sternum 2 covering sides of sternum 3 and 4 and pygidium exposed, protruding from the elytra. *Sibinia luteoviridis* (Gyllenhal, 1836) and *Sibinia micros* Caldara, 1989 were previously recorded in the vicinity of Gobabeb, in pitfall traps in the bed of the ephemeral Kuiseb river (Henschel et al. 2003). Further morphological analysis is required to check whether *Sibinia* sp. belongs to any of the two species known from the Kuiseb. According to Clark (1978), *Sibinia* species are exclusive plant feeders, and spend most of their life on the host plant.

**Hemiptera**

**Cicadomorpha**

**Cicadellidae**

**Deltocephalinae sp.**

A pale, flat and elongated leafhopper with a strongly spatulate crown (Figs S18–S20). The spatulate crown and hind leg chaetotaxy of the juveniles are fitting with Paradorydiini (according to Dmitriev 2001) but our specimens do not have the distinct median carina on the crown usually present in this group (Dmitriev 2001). For phylogenetic placement we retrieved 94 sequences of 28S rDNA of Deltocephalinae on genbank. Our species was found sister to the single Paradorydiini OTU: *Paradorydium lanceolatum* (Fig. S21). This genus is known from Namibia (*P. paradoxum* (Herrich-Schäffer, 1837) and *P. quadrigonum* (Naudé, 1926)), but none of the species matches the one presented here. We thus estimate our species to be a close relative of the genus *Paradorydium*, but remain careful with the taxonomic assignment due to the morphological discrepancy. *Paradorydium* spp. are commonly feeding on grasses, which is fitting with our species being collected on *S. sabulicola*.

**Coccomorpha**

**Diaspididae sp.**

This armoured scale insect was retrieved as a sister group of *Parlatoria* (2 spp on BOLD: *P. pergandii* and *P. theae*), with a bootstrap support of 30%. We consider that taxonomic assignment beyond the family is not currently possible with the barcode sequence. Morphological analysis will be required for genus and species determination. The species is found abundantly on the stem of *S. sabulicola*, encroached under the sheaths and is evidently a sap feeder. From the Sand Dune Sea, the Diaspididae *Namibia spinosa* Munting 1969 has been previously reported (Namibia National Committee for World Heritage 2012), but found on *Acacia* sp., but it seems that the Diaspididae on *Stipagrostis sabulicola* have never been formally identified. The honeydew produced by the Diaspididae associated with *Stipagrostis sabulicola* has been previously indicated as a food source for the desert dune ant *Camponotus detritus* (Curtis 2015).

**Pseudococcidae sp.**

From our sampling, all mobile individuals that resemble scale insects were devoid of silks. Some are probably the larvae of the Diaspididae sp. FPO. A larger specimen was picked for sequencing. Our phylogenetic placement recovered it nested within Pseudococcidae. Within the family, node bootstrap supports are generally extremely low. Our species is nested in a clade that regroups some *Phenacoccus* (paraphylatic), *Pseudococcus* (polyphyletic) and *Antonina*. We consider that taxonomic assignment beyond the family is not currently possible with the barcode sequence. Morphological analysis will be required.

**Hymenoptera**

**Chalcididae**

**Haltichellinae**

**Haltichellinae sp. FPO**

For this species, phylogenetic placement based purely on BOLD COI public data could not be achieved, due to the high level of retrieved paraphyly/polyphyly of and within the families of Parasitoid wasps. We retrieved all 18S rDNA, 28S D2D3 and 28S D5 sequences listed in the Chalcidoidea dataset of Heraty et al. (2013) (see their Appendix 1). Alignment was done with Muscle and performed a ML inference with Raxml-ng using the GTR+G model. We retrieved the species nested within Haltichellinae. Chalcididae associated with *Stipagrostis sabulica* were previously reported (Prinsloo, 1981).

**Psocodea**

**Liposcelidinae**

***Liposcelis* sp.**

Flat, wingless booklice (Fig. S28). Only a partial COI-5P could be retrieved from the sequencing data, with a reading frame broken in 5’. Nonetheless, the species is found nested within *Liposcelis* (Fig. S29) also fitting with the morphological habitus of the collected specimen. *Liposcelis* spp. have a mixed diet and can consume fungi and dead plant and animal material. On *S. sabulicola*, they seemed more abundant on the senescent and dead leaves.

**Thysanoptera**

**Phlaeothripidae**

***Haplothrips* sp.**

Black thrips (Fig. S30). After a rapid phylogenetic placement identifying it as a Phlaeothripidae, we retrieved all 28S sequences of this family on genbank and ran another analysis. Our species nested among *Haplothrips* species. We then retrieved all Haplothrips COI sequences on Genbank and BOLD and merged both sets. Our species was found sister of *Haplothrips cenchricola*, those species being nested in a strongly supported clade including several species of *Haplothrips* (Fig. S31). *Haplothrips nigricornis* and *H. tardus* have been reported from Namibia (NBD). Our species does not match with *H. nigricornis* represented on Genbank / BOLD *(H. tardus* not represented). Haplothrips species are polliniser, some are suspected to be predatory.

**FIGURES.**

**
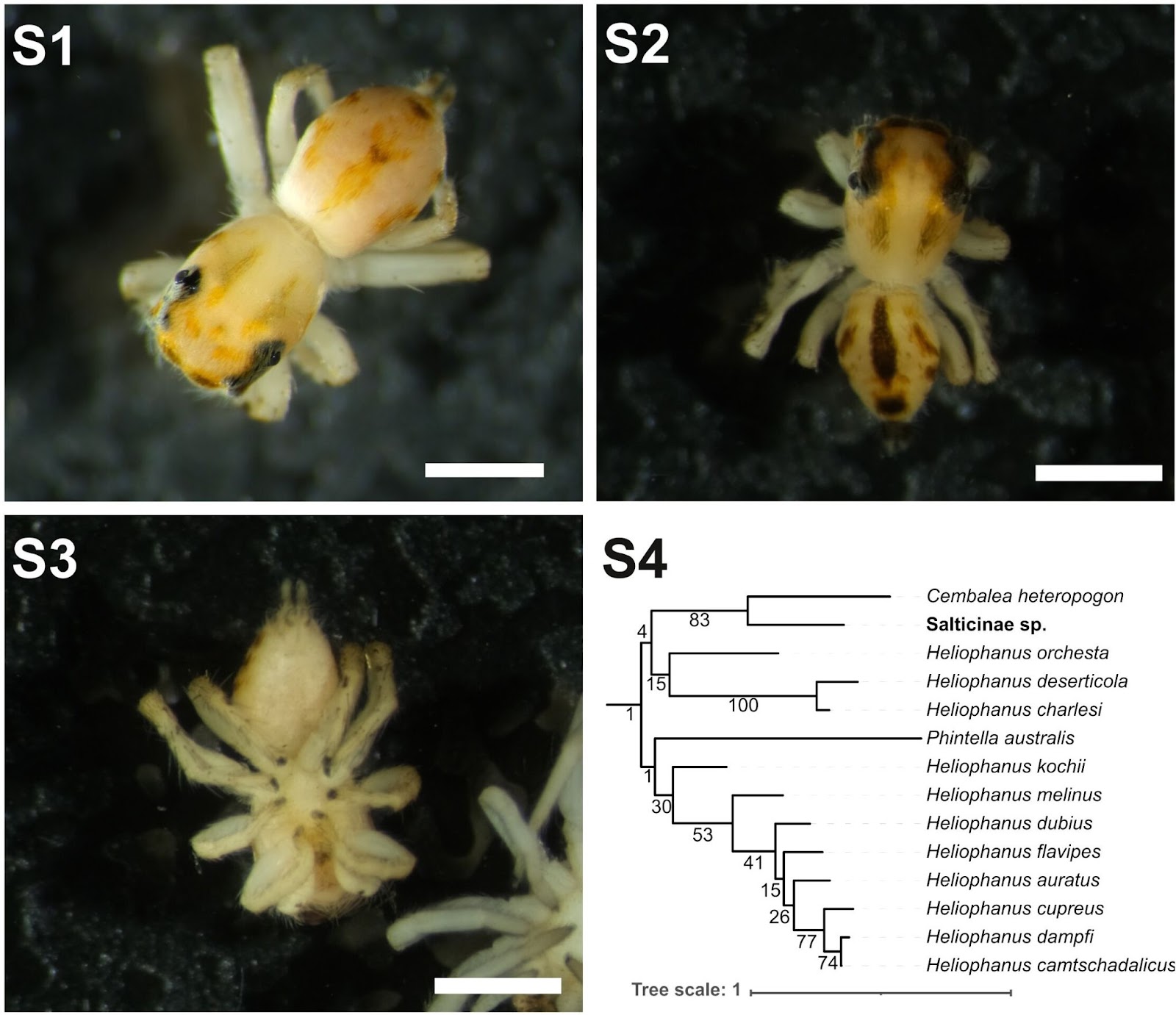
**

**Figures S1–4:** Salticinae sp. **S1** dorsal side, **S2** dorsal side colour variant, **S3** ventral side, **S4** phylogenetic placement among BOLD OTUs (COI-5P) using maximum likelihood criterion, bootstrap supports shown next to the nodes, our species shown in bold.


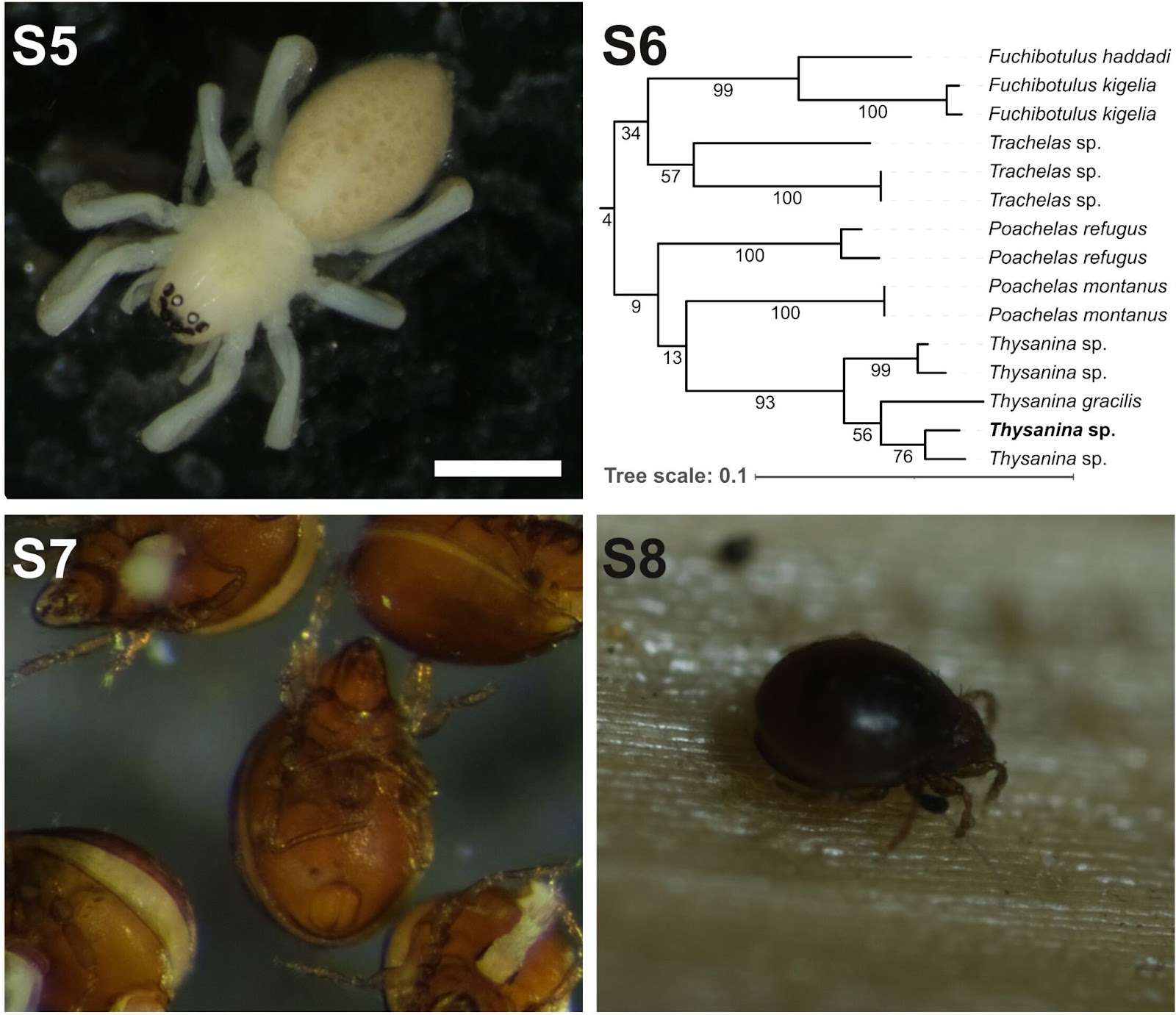


**Figures S5–S6:** *Thysanina* sp. **S5** dorsal side, **S6** phylogenetic placement among BOLD OTUs (COI-5P) using maximum likelihood criterion, bootstrap supports shown next to the nodes, our species shown in bold. **Figures S7–S8:** *Zygoribatula* sp. **S7** ventral side, **S8** on the stem of *S. sabulicola*.


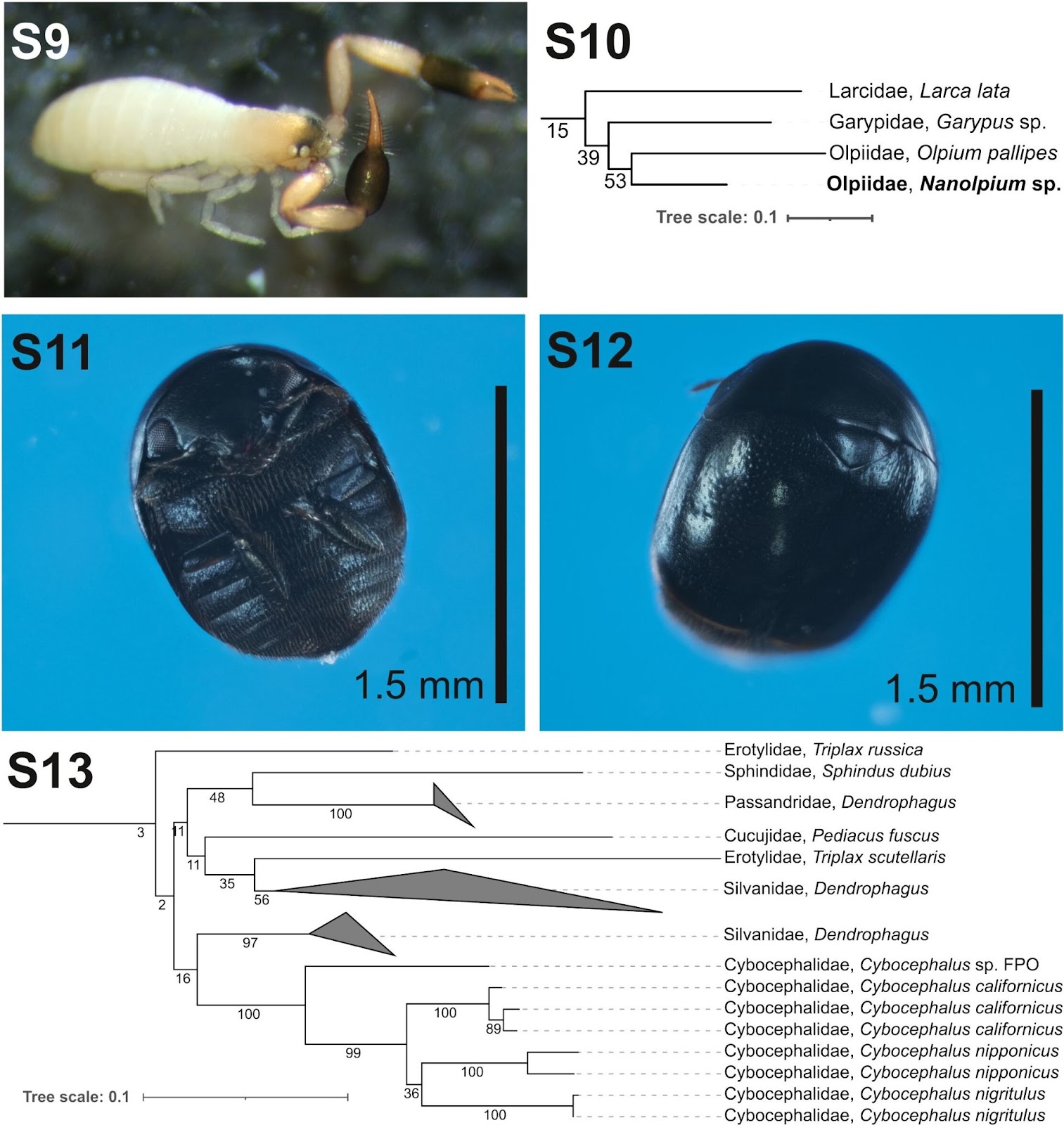


**Figures S9–S10:** *Nanolpium* sp. **S9** dorsal side, **S10** phylogenetic placement among BOLD OTUs (COI-5P) using maximum likelihood criterion, bootstrap supports shown next to the nodes, our species shown in bold. **Figures S11–S13:** *Cybocephalus* sp. **S11** dorsal side, **S12** ventral side; **S13** phylogenetic placement among BOLD OTUs (COI-5P) using maximum likelihood criterion, bootstrap supports shown next to the nodes, our species shown in bold.


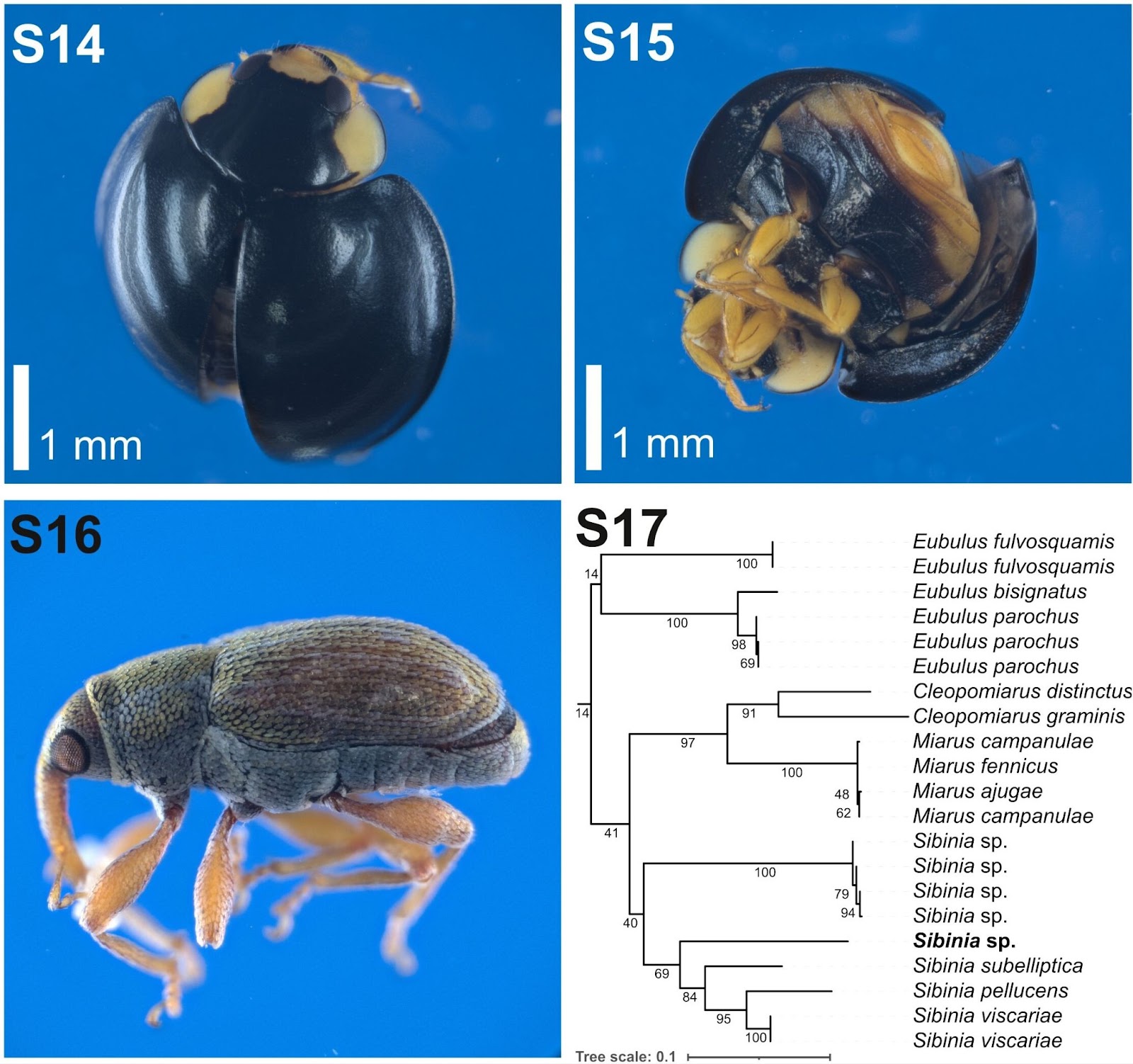


**Figures S14–S15:** *Exochomus flaviventris* **S14** dorsal side, **S15** ventral side.

**Figures S16–S17:** *Sibinia* sp. **S16** lateral side; **S17** phylogenetic placement among BOLD OTUs (COI-5P) using maximum likelihood criterion, bootstrap supports shown next to the nodes, our species shown in bold.


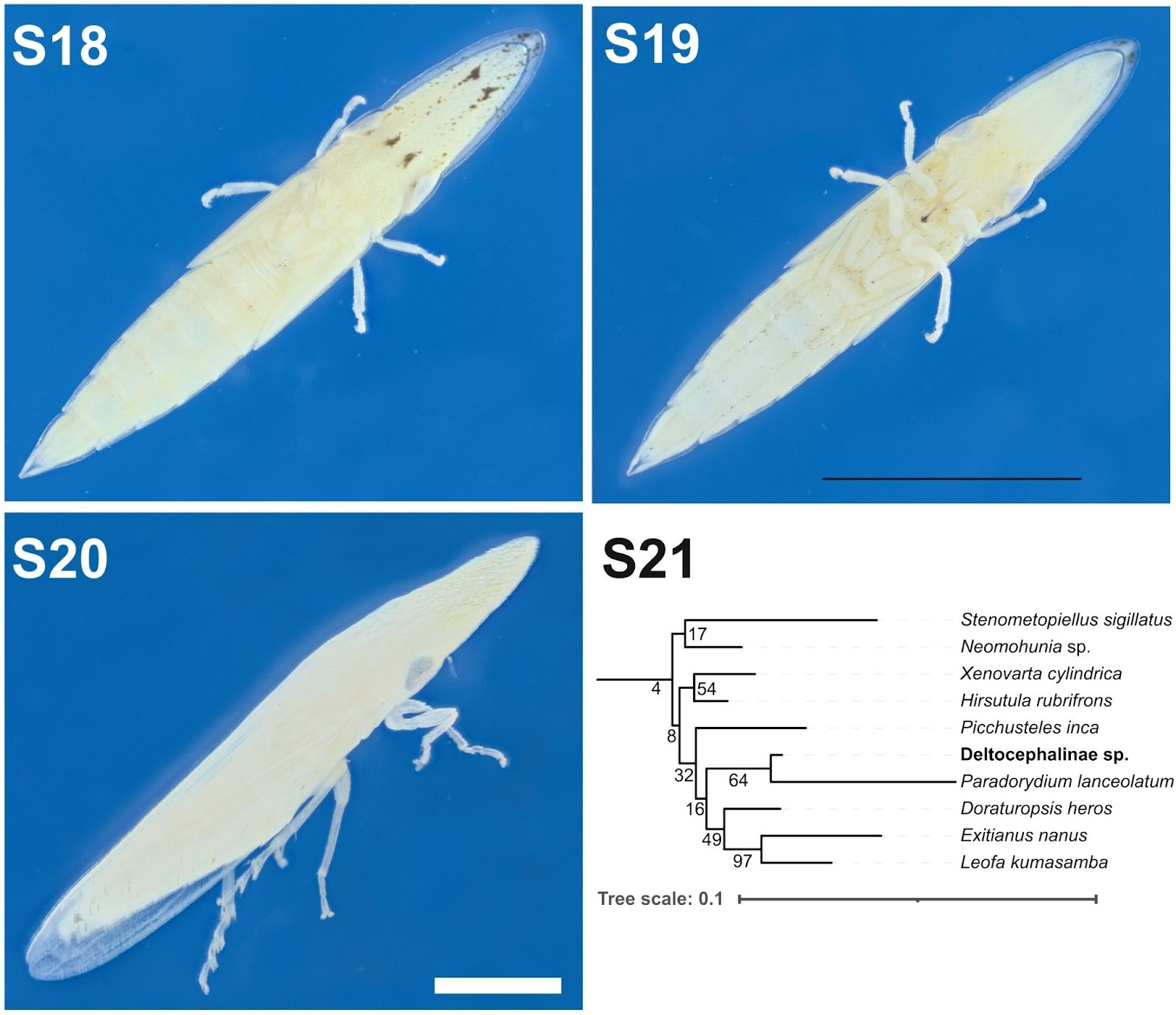


**Figures S18–S21:** Deltocephalinae sp. **S18** larva dorsal side, **S19** larva ventral side, **S20 adult**, dorso-lateral view, **S21** phylogenetic placement among BOLD OTUs (COI-5P) using maximum likelihood criterion, bootstrap supports shown next to the nodes, our species shown in bold.


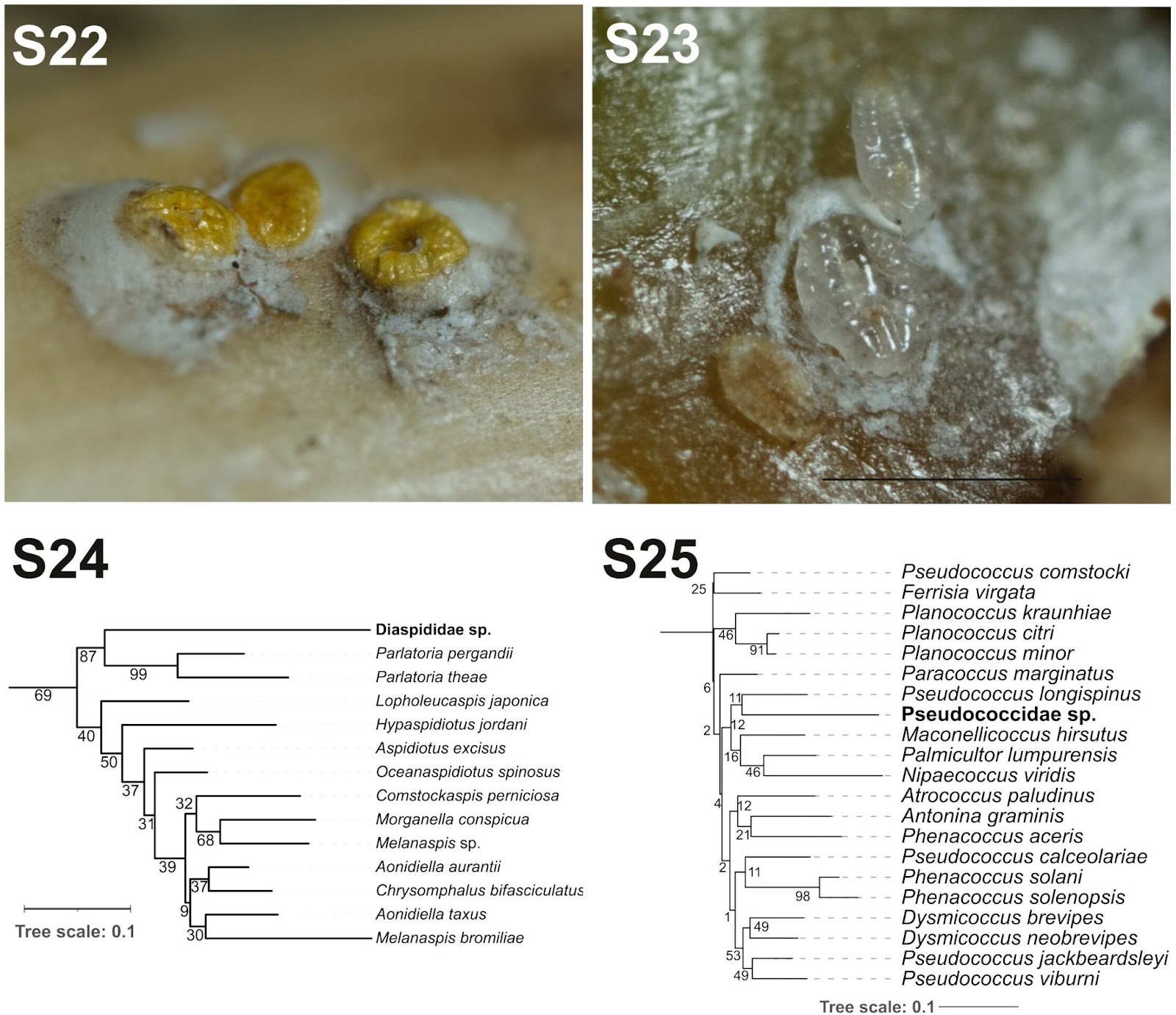


**Figures S22–S24:** Diaspididae sp. **S22** individuals on *S. sabulicola*, **S23** manually uncovered adult body, **S24** phylogenetic placement among BOLD OTUs (COI-5P) using maximum likelihood criterion, bootstrap supports shown next to the nodes, our species shown in bold. **Figure S25** : Pseudococcidae sp., phylogenetic placement among BOLD OTUs (COI-5P) using maximum likelihood criterion, bootstrap supports shown next to the nodes, our species shown in bold.


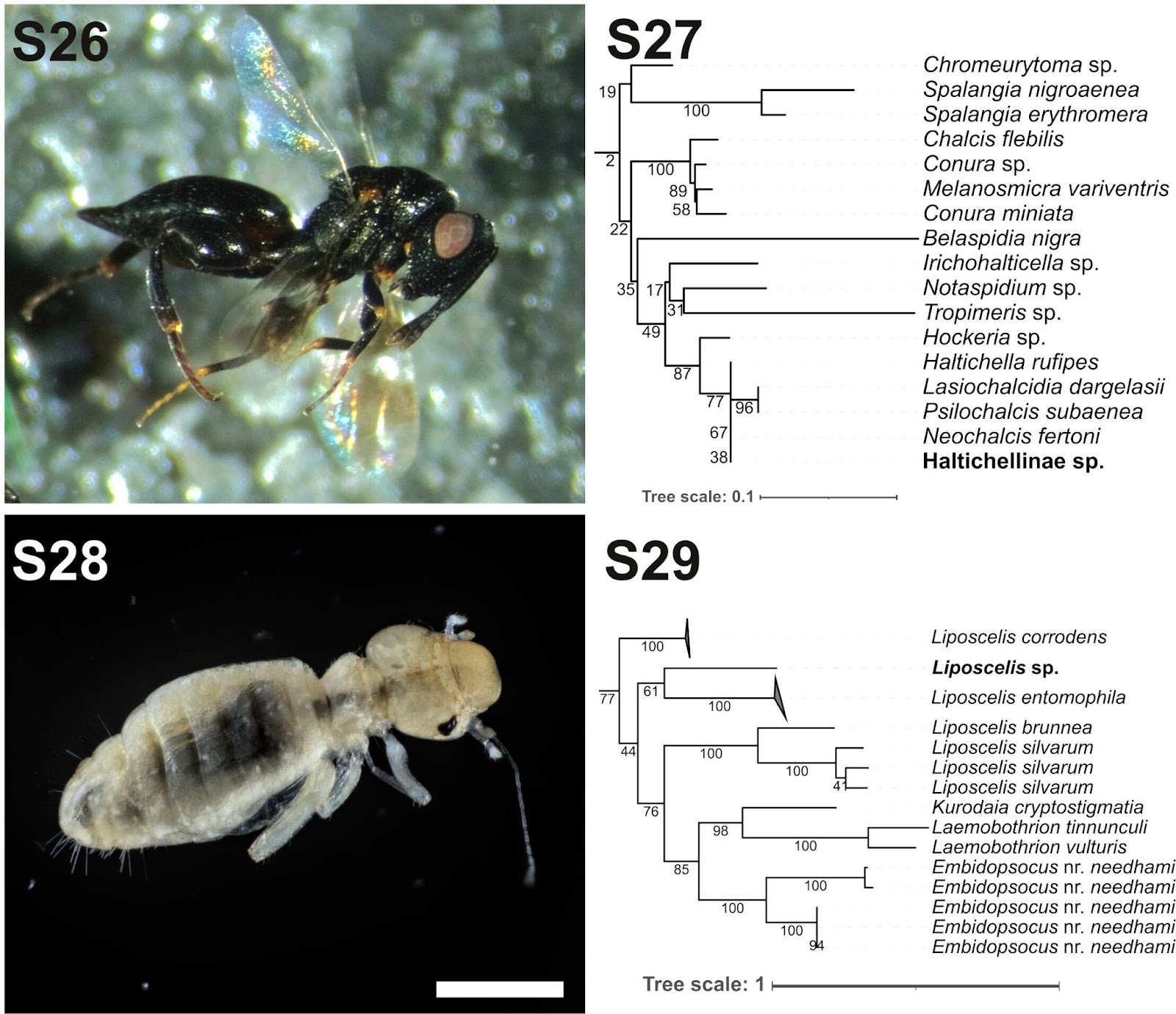

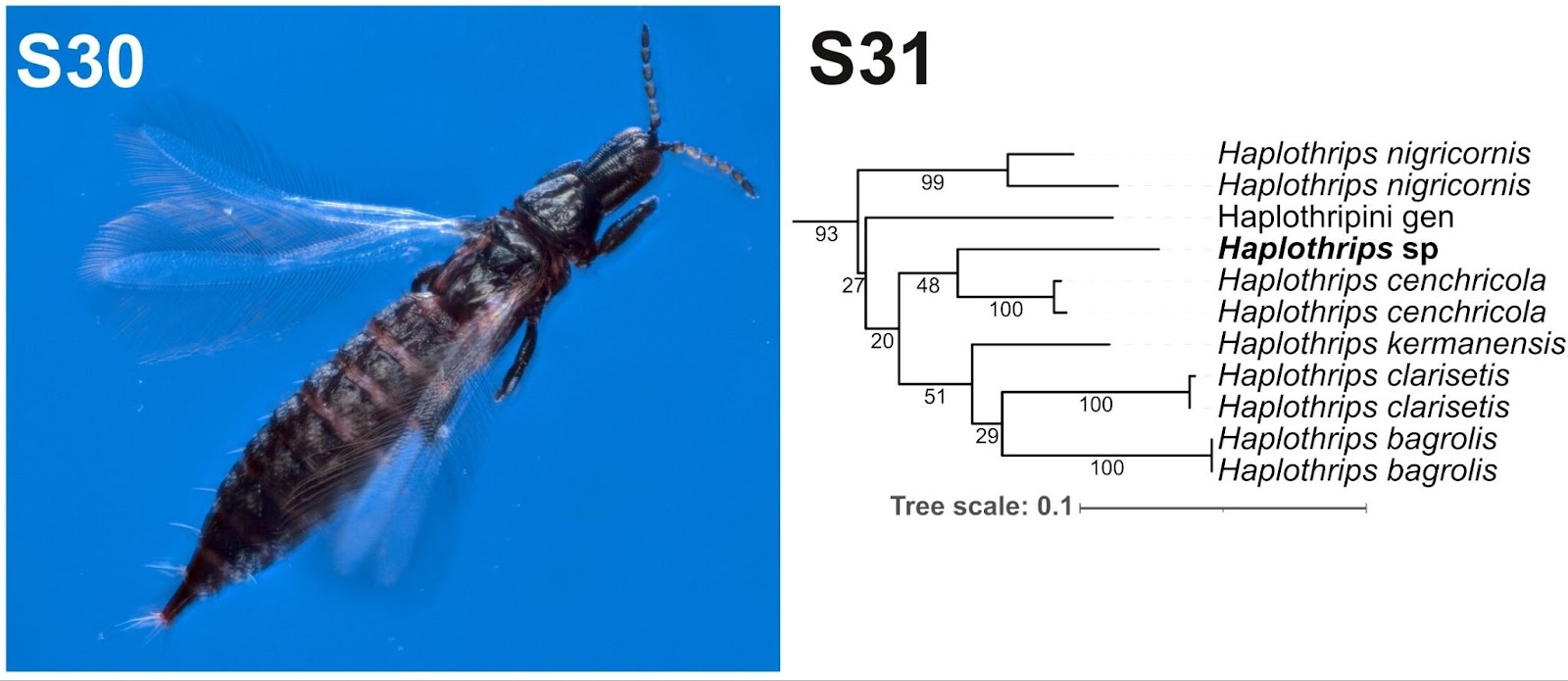


**Figures S26, S27:** Haltichellinae sp. **S26** lateral side, **S27** phylogenetic placement among Genbank OTUs (18S + 28S) using maximum likelihood criterion, bootstrap supports shown next to the nodes, our species shown in bold. **Figures S28, S29** : *Liposcelis* sp. **S28** dorsal side, **S29** phylogenetic placement among BOLD OTUs (COI-5P) using maximum likelihood criterion, bootstrap supports shown next to the nodes, our species shown in bold. **Figures S28, S29** : *Haplothrips* sp. **S30** dorsal side, **S31** phylogenetic placement among BOLD + Genbank OTUs (COI-5P) using maximum likelihood criterion, bootstrap supports shown next to the nodes, our species shown in bold.

**TABLE.**

**Table S1:** Genbank accession number for the COI-5P and 28S rDNA of the sequenced species.

| **Taxon** | **COI Accession number** | **28S Accession Number** |
| --- | --- | --- |
| Salticinae sp. | TO BE PROVIDED | TO BE PROVIDED |
| *Thysanina* sp. | TO BE PROVIDED | TO BE PROVIDED |
| *Nanolpium* sp. | TO BE PROVIDED | TO BE PROVIDED |
| *Zygoribatula* sp. | TO BE PROVIDED | TO BE PROVIDED |
| *Cyphobelus* sp. | TO BE PROVIDED | TO BE PROVIDED |
| *Sibina* sp. | TO BE PROVIDED | TO BE PROVIDED |
| Deltocephalinae sp. | TO BE PROVIDED | TO BE PROVIDED |
| Diaspididae sp. | TO BE PROVIDED | TO BE PROVIDED |
| Pseudococcidae sp. | TO BE PROVIDED | TO BE PROVIDED |
| Haltichellinae sp. | TO BE PROVIDED | TO BE PROVIDED |
| *Liposcelis* sp. | TO BE PROVIDED | TO BE PROVIDED |
| *Haplothrips* sp. | TO BE PROVIDED | TO BE PROVIDED |

**REFERENCES**

Beier M. (1964) Weiteres zur Kenntnis der Pseudoscorpioniden-Fauna des südlichen Afrika. *Annals of the Natal Museum* **16**, 30–90.

Beier M. (1966) Ergänzungen zur Pseudoscorpioniden-Fauna des südlichen Afrika. *Annals of the Natal Museum* **18**, 455–470.

Clark W. (1978) The weevil genus *Sibinia*: natural history, taxonomy, phylogeny, and zoogeography, with revision of the New World species. *Quaestiones entomologicae* **14** (2), 92–389.

Curtis B. A. (1985) Activity of the Namib Desert dune ant, *Camponotus detritus*. *South African Journal of Zoology* **20**, 41–48.

Dmitriev D. A. (2001) Larvae of some species of the subfamily Eupelicinae (Homoptera: Cicadellidae). *Zoosystemalica Rossica* **9** (2), 353–357.

Fabres G., Kiyindou A. and Epouna-Mouinga S. (1981) Les Entomophages inféodés à la cochenille du manioc *Phenacoccus manihoti* (Hom. Pseudococcidae) en République Populaire du Congo : 2 - étude morphologique comparative de trois espèces dominantes de Coccinellidae (Col.). *Cahiers ORSTOM. Série Biologie*, 3–8.

Fürsch H. (1961) Revision der afrikanischen Arten um *Exochomus flavipes* Thunb. Col. Cocc.. *Entomologische Arbeiten Museum G. Frey* **12**, 68–92.

Haddad C. R. and Wesołowska W. (2011) New Species and New Records of Jumping Spiders (Araneae: Salticidae) from Central South Africa. *African Invertebrates* **52** (1), 51–134.

Henschel J. R., Mtuleni V., Pallett J. and Seely K. M. (2003) The surface-dwelling arthropod fauna of Gobabeb with a description of the long-term pitfall trapping project. *Namibia Scientific Society* **51**, 65–92.

Heraty J. M., Burks R. A., Cruaud A., Gibson G. A. P., Liljeblad J., Munro J., Rasplus J.-Y., Delvare G., Janšta P., Gumovsky A., Huber J., Woolley J. B., Krogmann L., Heydon S., Polaszek A., Schmidt S., Darling D. C., Gates M. W., Mottern J., Murray E., Dal Molin A., Triapitsyn S., Baur H., Pinto J. D., van Noort S., George J. and Yoder M. (2013) A phylogenetic analysis of the megadiverse Chalcidoidea (Hymenoptera). *Cladistics* **29** (5), 466–542.

Irish J. (2002-2023) Namibia Biodiversity Database Web Site, https://www.biodiversity.org.na, accessed the 15th of October 2023.

Judson M. L. I. and Heurtault J. (1996) *Nanolpium* species (Garypoidea, Olpiidae) on grasses in southern Africa - a new niche for pseudoscorpions." Proceedings of the XIIIth Congress of Arachnology, 3-8 September 1995, Geneva. *Revue Suisse de Zoologie* **HS**, 321–326.

Kanika-Kiamfu J., Iperti G. and Brun J. (1993) Étude de la consommation alimentaire d'*Exochomus flaviventris* (Col.: Coccinellidae) prédateur de la cochenille du maniocPhenacoccus manihoti (Hom.: Pseudococcidae). *Entomophaga* **38**, 291–298.

Lyle R. and Haddad C. R. (2006) A revision of the Afrotropical tracheline sac spider genus *Thysanina* Simon, 1910 (Araneae: Corinnidae). *African Invertebrates* **4**7, 95–116.

Prinsloo, G. L. (1981). On the encyrtid parasites (Hymenoptera: Chalcidoidea) associated with psyllids (Hemiptera: Psylloidea) in southern Africa. *Journal of Entomological Society of Southern Africa*, *44*, 199–244.

Seely M. (Ed.), (2012) *Namib Sand Sea World Heritage Nomination.* *Annexes*. Namibia National Committee for World Heritage, Windhoek, 1–496.

Smith T. (2022) Review of the Cybocephalidae (Coleoptera) of North America and the West Indies with descriptions of two new species of 𝐶𝑦𝑏𝑜𝑐𝑒𝑝ℎ𝑎𝑙𝑢𝑠 Erichson. *Insecta Mundi* **0950**, 1–35.

Umeh E.-D. N. (1990) *Exochomus troberti* Mulsant (Coleoptera: Coccinellidae): A predator of cassava mealybug, *Phenacoccus manihoti* Mat-Ferr (Homoptera: Pseudococcidae) in southeastern Nigeria. *International Journal of Tropical Insect Science* **11** (02), 189–195.

Wesołowska W. (2011) New species and new records of jumping spiders from Botswana, Namibia and Zimbabwe (Araneae: Salticidae). *Genus* **22** (2), 307–346.
